# Molybdenum and Vanadium-Codoped Cobalt Carbonate Nanosheets Deposited on Nickel Foam as a High-Efficient Bifunctional Catalyst for Overall Alkaline Water Splitting

**DOI:** 10.3390/molecules29153591

**Published:** 2024-07-30

**Authors:** Wenxin Wang, Lulu Xu, Ruilong Ye, Peng Yang, Junjie Zhu, Liping Jiang, Xingcai Wu

**Affiliations:** 1 State Key Laboratory of Analytical Chemistry for Life Science, Nanjing University, Nanjing 210023, China; 2 School of Chemistry and Chemical Engineering, Nanjing University, Nanjing 210023, China; 3 Key Laboratory of Mesoscopic Chemistry of MOE, Nanjing University, Nanjing 210023, China

**Keywords:** Mo and V-codoped CoCOx nanosheets, bifunctional electrocatalyst, alkaline water splitting, DFT calculation, response surface methodology

## Abstract

To address issues of global energy sustainability, it is essential to develop highly efficient bifunctional transition metal-based electrocatalysts to accelerate the kinetics of both the hydrogen evolution reaction (HER) and the oxygen evolution reaction (OER). Herein, the heterogeneous molybdenum and vanadium codoped cobalt carbonate nanosheets loaded on nickel foam (VMoCoCOx@NF) are fabricated by facile hydrothermal deposition. Firstly, the mole ratio of V/Mo/Co in the composite is optimized by response surface methodology (RSM). When the optimized composite serves as a bifunctional catalyst, the water-splitting current density achieves 10 mA cm^−2^ and 100 mA cm^−2^ at cell voltages of 1.54 V and 1.61 V in a 1.0 M KOH electrolyte with robust stability. Furthermore, characterization is carried out using field emission scanning electron microscopy-energy dispersive spectroscopy (FESEM-EDS), high-resolution transmission electron microscopy (HRTEM), X-ray diffraction (XRD), and X-ray photoelectron spectroscopy (XPS). Density functional theory (DFT) calculations reveal that the fabricated VMoCoCOx@NF catalyst synergistically decreases the Gibbs free energy of hydrogen and oxygen-containing intermediates, thus accelerating OER/HER catalytic kinetics. Benefiting from the concerted advantages of porous NF substrates and clustered VMoCoCOx nanosheets, the fabricated catalyst exhibits superior electrocatalytic performance. This work presents a novel approach to developing transition metal catalysts for overall water splitting.

## 1. Introduction

Recently, increasing populations and rapid consumption of fuel for industrial and agricultural production have boosted the energy crisis [1]. Electrochemical water electrolysis is an important strategy to convert water to hydrogen (H_2_) and oxygen (O_2_) in today’s need for clean and recyclable energy that has a high capability for storage [1]. H_2_ is one of the most important clean energy fuels, which can be used in fuel cells to produce electricity or as a fuel gas in heating devices. For large-scale H_2_ production, electrochemical water splitting offers the dual benefits of sustainable clean energy generation and an effective reduction in carbon discharge. Highly effective electrocatalysts are required in water electrolysis to accelerate the kinetics of hydrogen and oxygen evolution (HER and OER). Currently, noble metal-based materials are promising catalysts for use in electrochemical water splitting [2]. Platinum (Pt) is the best-ever high-efficiency HER catalyst, while IrO_2_ and RuO_2_ are deemed the best-known OER catalysts. However, due to their high cost and scarcity, these materials lack the feasibility of developing scalable electrocatalysts for new energy production [3,4]. To this end, it is urgent to develop durable, cheap, and efficient electrocatalysts from earth-abundant transition metals, which is vital for the development of the H_2_ economy [5].

Transition metal-based electrocatalysts, due to their Earth-abundant resources, cheapness, and comparable catalytic performance, have attracted enormous interest as alternatives to expensive and scarce noble metals [5]. However, their HER performances are not satisfactory under alkaline media, and the excessively high Gibbs energy barriers (ΔG) limit their HER activity. The OER suffers from more sluggish charge/electron transfer kinetics because the OER is a four-step charge/electron transfer, resulting in a high requirement for electrocatalysts. Therefore, developing cheap, stable, and active bifunctional electrocatalysts for the achievement of highly efficient electrochemical H_2_ production is challenging. For this purpose, transition metals/transition metallic compounds are introduced as potential materials for developing highly efficient electrocatalysts for water electrolysis. Transition metallic bifunctional electrocatalysts are advantageous owing to their special electronic structures of atoms. The electronic states of bimetallic/multimetallic catalysts can be tuned by heteroatom codoping [6,7] or oxygen vacancies [8]. Generally, heteroatom codoping is an effective approach for bifunctional non-noble metal catalysts to accelerate charge/electron transfer and expose more active sites.

Compared with expensive and scarce noble metals or noble metallic compounds, the lower band gaps and stronger electron-donating abilities of transition metals such as vanadium (V), molybdenum (Mo), and cobalt (Co) make them excellent dopant candidates to enhance catalytic activity [5]. Their electrocatalytic activities are mainly dependent on their electronic structures, active sites, charge/electron transfer, and surface energy/surface area [9]. The co-element has attracted great attention for its ability to accelerate charge/electron diffusion, resulting in high efficiency in overall water splitting [10]. Mo-based electrocatalytic materials have been fabricated as efficient HER and OER catalysts in alkaline media due to their multiple valence states and unique redox properties. V element with multiple valence states, rich earth abundance, and high electrochemical performance is a promising OER dopant to effectively tune the synergistic effect among different metal active sites [11]. Zhou et al. fabricated V-doped Ni_3_S_2_@NiFe LDH ultrathin nanosheets on porous Ni foam (NF) substrates as a highly effective electrocatalyst for water splitting. The presence of V guarantees a remarkable promotion of electrocatalytic kinetics and activity [12]. Yet, the intrinsic performance of HER was still inferior due to the weak Gibbs free energy change in hydrogen adsorption (ΔG_H*_) on the V atom [8]. The addition of transition metals can modify the electronic structure of electrocatalysts, thus exposing more active sites. Xiao et al. fabricated the MoCo(OH)_2_/CoP@NF, which exhibited enhanced catalytic activity toward the OER [13]. Yan et al. combined Co with Mo_2_C, which could decrease the ΔG_H*_, thus improving the catalytic activity [14]. Inspired by the above outcomes, multi-transition-metal composites can synergistically optimize the HER/OER catalytic activity. Doping high-valence V and Mo ions can optimize the electronic structure of transition metal materials owing to their electron-drawing capacity. The nanosheet structure of cobalt carbonate facilitates ion/electron diffusion and exposes more active sites. Moreover, porous NF is an intriguing substrate for enhancing electronic states and active sites due to its porous surface area [15]. To the best of our knowledge, the development of multimetallic codoped bifunctional electrocatalysts loaded on porous NF substrates for bifunctional water splitting has rarely been reported till now.

**Scheme 1:**
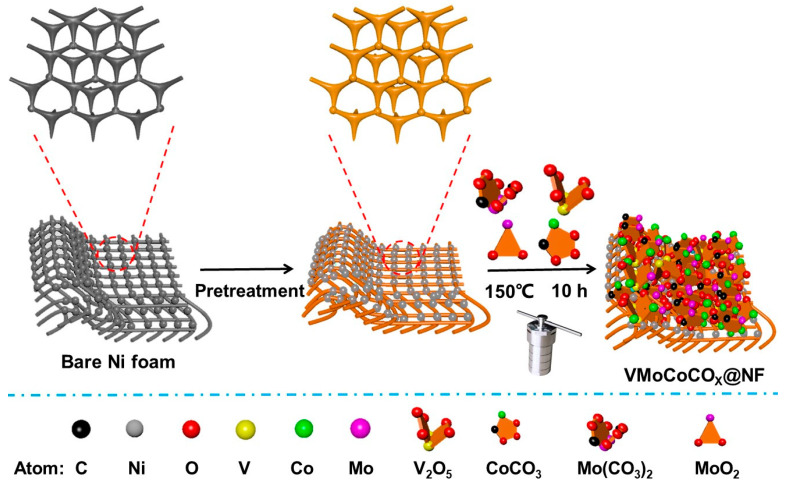
Schematic diagram for the fabrication procedures of VMoCoCOx@NF.

In this work, the V and Mo-codoped CoCOx nanosheets in situ loaded on a porous NF substrate (VMoCoCOx@NF) were fabricated via facile hydrothermal deposition (Scheme 1). Firstly, response surface methodology (RSM) was employed to achieve the optimum mole ratio of V/Mo/Co in the catalyst. More detailed illustrations of RSM, as well as related data graphs (Figures S1 and S2), were given in the Supporting Information (SI). The predicted RSM model responses of VMoCoCOx@NF were well-fitted for all the actual responses (Figure S2). The optimized catalyst showed remarkable HER/OER activities in a 1.0 mol L^−1^ (M) KOH electrolyte. Meanwhile, density functional theory (DFT) was used to fully illustrate the high HER/OER activities of the fabricated catalyst. Additionally, the excellent HER/OER activities for VMoCoCOx@NF enabled it to serve as both anode and cathode for the alkaline electrolyzer, a catalyst that required cell voltages of 1.54 V/1.61 V to reach current densities of 10 mA cm^−2^/100 mA cm^−2^ with excellent cycling stability.

## 2. Results and Discussion

### 2.1. Characterizations of Catalysts

**Figure 1:**
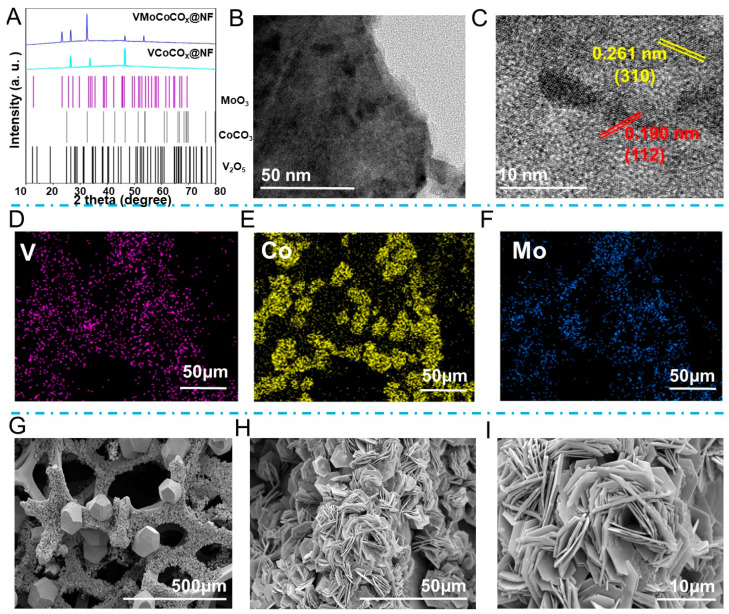
(**A**) XRD patterns of VCoCOx@NF (lake blue), VMoCoCOx@NF (navy blue), and references of pure MoO_3_, CoCO_3,_ and V_2_O_5_. Notably, the diffraction lines of Ni were removed during the XRD operation. (**B**) TEM and (**C**) HRTEM images of the NF substrate and VMoCoCOx@NF, respectively. EDS elemental mapping images of V (**D**), Co (**E**), and Mo (**F**) for the surface of VMoCoCOx@NF. FESEM images of VMoCoCOx@NF scanned at 500 μm (**G**), 50 μm (**H**), and 10 μm scales (**I**).

The structure of the VMoCoCOx@NF was confirmed by X-ray diffraction (XRD) and high-resolution transmission electron microscopy (HRTEM). The crystallinity of the catalysts was characterized by the XRD technique within the 2θ range of 10–80°. The XRD patterns of VMoCoCOx@NF and VCoCOx@NF and the XRD positions for pure MoO_3_, CoCO_3,_ and V_2_O_5_ references were depicted in Figure 1A. The strong diffraction lines at 44.2°, 51.8°, and 76.3° correspond to the standard pattern of Ni (PDF#04-0850) in NF substrate. For comparison, the XRD positions for pure MoO_3_, CoCO_3,_ and V_2_O_5_ references were indicated according to the PDF# database. In Table S7, the diffraction lines at 19.9°, 26.5°, 30.8°, 33.7°, 47.1°, and 50.8° correspond to V_2_O_5_ (PDF#00-041-1426), the diffraction lines at 23.3°, 25.7°, and 27.3° are well indexed to MoO_3_ (PDF#05-0508), and the diffraction lines at 42.9°, 46.5°, 51.8°, and 53.5° are attributed to CoCO_3_ (PDF#11-0692). The correspondence with MoO_3_ marked on the picture was observed at 23.3°, which corresponds to MoO_3_ (PDF#05-0508). Importantly, in Figure 1A and Figure S3, the diffraction lines of Ni were removed during the XRD operation. The diffraction lines of VMoCoCOx@NF were observed at approximately 23.3°, 26.5°, 32.5°, 46.5°, and 53.5°. The diffraction lines of VCoCOx@NF were observed at approximately 26.5°, 33.7°, and 46.5°, indicating that the characteristic lines of MoO_3_, V_2_O_5,_ and CoCO_3_ were visible at 23.3°, 26.5°, and 46.5° in the synthesized hybrid catalyst VMoCoCOx@NF. The difference was that the additional line towards the diffraction angle of 32.5° was for VMoCoCOx@NF, indicating the formation of a new phase (112) [16]. It suggests that, compared with VCoCOx@NF, an appropriate amount of Mo was assembled into the hybrid. The corresponding diffraction line at 32.5° might arise from the doping of Mo.

To further analyze the morphology of the sample, the TEM images in Figure 1B show crystalline nanosheets embedded in the NF substrate. The portions with agglomerates should be the nano-edge structure of NF. The HRTEM image of VMoCoCOx@NF reveals the well-resolved lattice fringes of 0.190 nm and 0.261 nm, which are in agreement with the (310) and (112) planes of VMoCoCOx@NF (Figure 1C and Figure S4A) [17], which is also in agreement with the XRD observation in Figure 1A. Notably, Figure S4B,C also clearly exhibits that the accurate measured values of 10 neighbor lattice fringes in the corresponding crystal plane are 1.90 nm and 2.61 nm, which are exactly ten times the single d-spacing values measured by HRTEM. The crystal structure of the VMoCoCOx@NF catalyst was constructed via strong coordination bond interaction among V, Mo, and Co; the lamellas were combined by an electrostatic force. Taking advantage of the synergistic effects of 3d and 4d vacancies and V/Mo codoping, the doped nanostructure was effectively modulated, and more active sites were exposed.

Moreover, the field emission scanning electron microscopy-energy dispersive spectroscopy (FESEM-EDS) elemental mapping images in Figure 1D–F validate the distribution of V, Mo, and Co elements over the surface of VMoCoCOx@NF. The EDS mappings indicate that the structure was mainly determined by CoCO_3_ since V_2_O_5_ and MoO_3_ were comparably more randomly distributed on the NF. Figure 1G–I characterize the morphology and nanostructure of the fabricated VMoCoCOx@NF catalyst by FESEM. The clustered hexagonal VMoCoCOx nanosheets were anchored on the surface of NF. It presented an interconnected porous architecture with abundant charge/electron transfer channels. From the FESEM image of VMoCoCOx@NF (Figure 1G), it can be seen that a small number of nanoparticles, that is, CoCO_3_ (K_sp_ = 1.4 × 10^−13^), are deposited onto the VMoCoCOx nanosheet surface, further being confirmed by the Fourier transform infrared spectrum (FTIR) of VMoCoCOx@NF (Figure S5). FTIR spectroscopy was utilized to further identify the functional groups on the surface of VMoCoCOx@NF, VCoCOx@NF, and V_2_O_5_@NF. The FTIR spectrum of VMoCoCOx@NF shows that the peaks at 1667.00 cm^−1^, 1078.15 cm^−1^, and 833 cm^−1^ are assigned to the stretching vibration of the C=O bond, the C-O bond, and the bending vibration of the CO_3_^2−^ functional group. The peak around 1100 cm^−1^ is distinctive for VMoCoCOx@NF. The peak at 478.7 cm^−1^ is attributed to the vibration of the VO_4_^3−^ functional group [18]. The FTIR measurement confirms the CO_3_^2−^ existing in the hybrid catalyst VMoCoCOx@NF. The FESEM image in Figure 1H demonstrates clustered hexagonal ultrathin nanosheet morphology with a thickness of approximately 100 nm. Notably, such a unique structure provides irregular charge/electron transfer channels and an abundant porous architecture, which is not only beneficial to the diffusion/penetration of electrolytes and the escape of H_2_ and O_2_ but also offers a large surface area with abundant exposed active sites for reaction intermediates.

**Figure 2:**
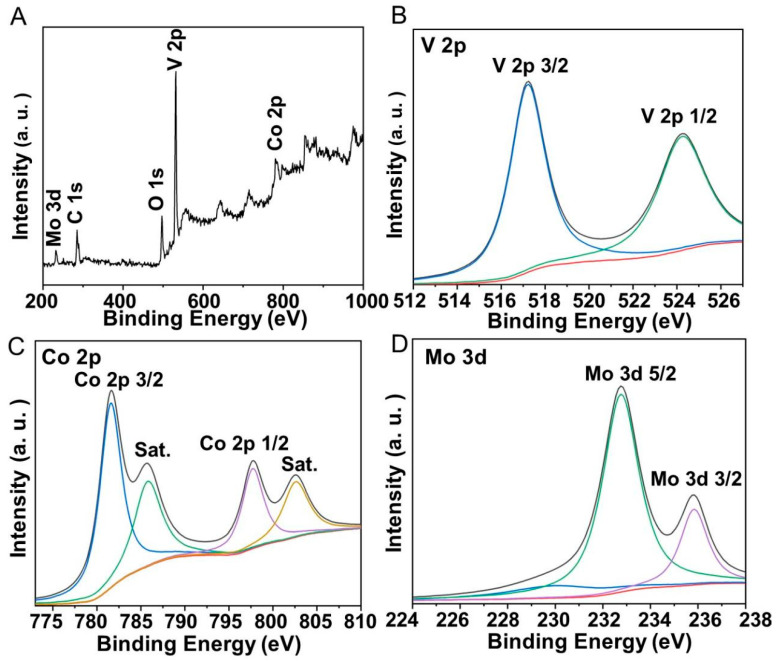
(**A**) XPS survey for VMoCoCOx@NF. High-resolution XPS spectra of (**B**) V 2p, (**C**) Co 2p, and (**D**) Mo 3d for VMoCoCOx@NF.

The full survey X-ray photoelectron spectroscopy (XPS) spectra of V 2p, Co 2p, and Mo 3d for the fabricated VMoCoCOx@NF catalyst are shown in Figure 2A, confirming the main peaks and valence states of V, Mo, and Co elements. The Ni peak was removed during the XPS operation. In the analysis of the XRD results shown in Figure 1A, all the nickel peak intensities were very large. Considering that a large amount of Ni from the NF substrate could be easily introduced into the hydrothermal system, the Ni peak was omitted during the XPS scanning process due to the visualization effect of the target peaks. Figure 2B shows the V2p spectrum; the two peaks were fitted into V 2p3/2 and V 2p1/2 located at 516.9 eV and 524.4 eV with a spin–orbit splitting of 7.5 eV, corresponding to the occurrence of V^+5^ (VO_4_^3−)^ species. The V on the surface mainly existed in the V^5+^ species. The high-valence V^5+^ species is crucial to higher electric conductivity, which benefits the improvement of catalytic performance, which is consistent with previous works [19,20]. In the XPS diagram of the 2p orbital of Co (Figure 2C), 2p1/2 and 2p3/2 only deconvolve one peak. The major peaks of 781.7 and 782.1 eV are consistent with the deconvolution of Co 2p 3/2. Simultaneously, peaks of 796.4 and 797.09 eV conform to the spin–orbit characteristics of Co2p 1/2, and the two vibrating satellite peaks are observed at 786.23 eV and 802.61 eV for high-resolution Co 2p spectra [21]. Interestingly, the spin–orbit splitting of about 16 eV between Co 2p 3/2 and Co 2p 1/2 peaks is observed to prove the Co^2+^ species in VMoCoCOx@NF [10,17]. The Mo doping caused an upshift of Co 2p binding energy in VMoCoCOx@NF, indicating that the electronic structure of Co was greatly adjusted by the charge transfer from the electron cloud around Co to the doped atoms [4]. It is essential to verify the efficient doping of the elements and evaluate a possible synergistic effect that might influence HER/OER activity [22]. Figure 2D shows the Mo 3d region XPS spectrum for VMoCoCOx@NF, in which only two peaks of Mo 3d5/2 and Mo 3d3/2 at 232.5 eV and 235.6 eV were identified and assigned to Mo^6+^ (MoO_4_^2−^). However, the peaks at ~228 eV and 232 eV, which correspond to lower bonding energies of Mo^4+^ (3d5/2 and 3d3/2), were not observed, indicating that Mo^4+^ did not exist in the catalyst [23]. In Figure 1A, VMoCoCOx@NF has a small diffraction peak at 23.3°. The XRD analysis shows the presence of MoO_3_ (PDF#05-0508). This result is also consistent with the result of the XPS analysis (Figure 2D), confirming the presence of only Mo^6+^ (MoO_4_^2−^) species. The emergence of the MoO_4_^2−^ peaks suggested that Mo was doped into the catalyst. Doping high-valence Mo^6+^ and V^5+^ ions into CoCO_3_ altered the electronic structure of the 3d metal atom, owing to the capability of Mo^6+^ and V^5+^ to draw electrons. The XPS and FTIR results confirmed the MoO_4_^2−^, VO_4_^3−^, Co^2+^, and CO_3_^2−^ species presented in VMoCoCOx@NF. The synergistic effect should help elucidate the atomic interactions among V, Mo, and Co.

### 2.2. Electrocatalytic HER Performance

**Figure 3:**
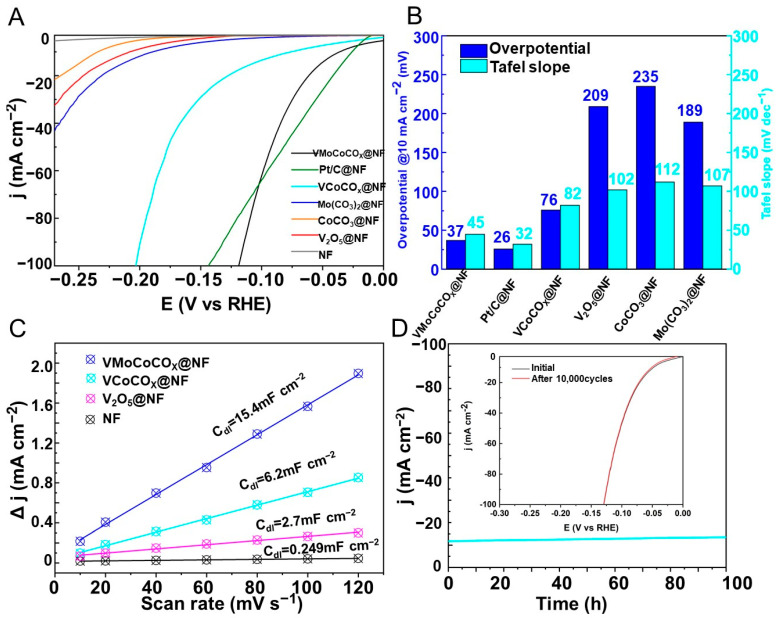
Electrocatalytic properties of the catalysts selected for HER. (**A**) LSV polarization curves. (**B**) Comparison of Tafel slopes and overpotentials of the catalysts at a constant *j* = −10 mA cm^−2^. (**C**) Estimation of C_dl_ by plotting the ratios of catalyst Δ*J* to scan rate. (**D**) Durability test of VMoCoCOx@NF performed at a constant *j* = −10 mA cm^−2^ within 100 h. The inset showed the LSV curves initially and after 10,000 CV cycles.

The electrocatalytic HER performance of the as-prepared electrocatalysts was measured using the standard three-electrode system in the N_2_-saturated 1.0 M KOH electrolyte. For comparison, the HER activities of Pt/C@NF, VCoCOx@NF, MoCO_3_@NF, CoCO_3_@NF, V_2_O_5_@NF, and NF were studied. Figure 3A displays the HER linear sweep voltammetry (LSV) curves of all samples recorded with a scan rate of 5 mV s^−1^ from 0.00 mV to −100 mV. As expected, the VMoCoCOx@NF catalyst displayed excellent alkaline HER activity, which agreed with the previous reports [17,22] (Table S8). The overpotential and Tafel slope are the most important parameters for clarifying the reaction kinetics of catalysts. Doping of two or more elements (salts) could efficiently facilitate the abundance of active sites, which resulted in high HER activity and significantly reduced the overpotential and Tafel slope [24]. To further obtain a clear comparison of the as-prepared catalysts, overpotentials and Tafel slopes required to reach a current density of 10 mA cm^−2^ were plotted in Figure 3B. Obviously, the overpotential of VMoCoCOx@NF was only 37 mV, which was much lower than that of VCoCOx@NF (76 mV), V_2_O_5_@NF (209 mV), CoCO_3_@NF (235 mV), and Mo(CO_3_)_2_@NF (189 mV). Nevertheless, the overpotential of commercial Pt/C@NF (26 mV) was found to be lower than that of VMoCoCOx@NF. This fact can only be explained by the highest HER activity of VMoCoCOx@NF among the as-prepared catalysts, except for Pt/C@NF. Additionally, the Tafel slopes for VMoCoCOx@NF, Pt/C@NF, VCoCOx@NF, V_2_O_5_@NF, CoCO_3_@NF, and Mo(CO_3_)_2_@NF were 45, 32, 82, 102, 112, and 107 mV dec^−1^, respectively. Figure S7 provides corresponding Tafel plots. We fitted the linear regions in the LSV curves of all of the samples to the Tafel equation to measure the corresponding Tafel slopes. As revealed in Figure S7A, the VMoCoCOx@NF exhibited a low Tafel slope of 45 mV dec^−1^ at the current density of 10 mA cm^−2^ during HER. The lower the Tafel slope, the faster the HER catalytic kinetics will be. The lowest Tafel slope of VMoCoCOx@NF among the catalysts also further proved that the grafting of V/Mo clustered nanosheets favorably promoted the HER catalytic kinetics. The electrochemical double layer capacitance (C_dl_) test was carried out to further evaluate the HER activity [23]. The cyclic voltammetry (CV) curves of VMoCoCOx@NF, VCoCOx@NF, and V_2_O_5_@NF recorded at various scan rates (10, 20, 40, 60, 80, 100, and 120 mV s^−1^) are displayed in Figure S8A–C. For better interpretation and comparison of electrochemical surface areas (ECSAs), we measured the C_dl_ behavior of the catalysts at the same scan rates. At each scan rate, the VMoCoCOx@NF catalyst provided much higher anodic (*i*a) and cathodic (*i*c) current densities (CV areas) than those of the other catalysts, consistent with a much greater active surface area. From these linear plots in Figure 3C or Figure S8D, we obtained the C_dl_ values of VMoCoCOx@NF, VCoCOx@NF, and V_2_O_5_@NF, and the bare Ni foam of 15.4, 6.2, 2.7, and 0.249 mF cm^−2^, respectively. The considerably higher C_dl_ of the VMoCoCOx@NF suggested that it had the highest ECSA and, correspondingly, the highest surface roughness when compared with those of the other catalysts. Furthermore, an electrochemical impedance spectroscopy (EIS) plot is collected to assess the charge transfer features of materials between the electrode and electrolyte interface. As illustrated in Figure S6, the VMoCoCOx@NF electrode exhibits a smaller radius than those of VCoCOx@NF, CoCOx@NF, and V_2_O_5_@NF, implying the smallest charge transfer resistance (R_ct_) for the HER process, suggesting it has excellent electron transfer. Above all, the Mo modification accelerates charge transfer in the HER process, and the higher HER activity of the VMoCoCOx@NF electrode can be explained by the smallest Tafel slope and minimal R_ct_.

Stability is another key parameter for the catalyst in practical applications. The stability of VMoCoCOx@NF was measured by CV and chronoamperometry (*i–t*) methods for HER at a constant current density of 10 mA cm^−2^ [10]. As displayed in Figure 3D, the stability of VMoCoCOx@NF was evidenced for more than 100 h, with the current density at about 93.7% of its original value, indicating an outstanding stability of the use of VMoCoCOx@NF. The inset in Figure 3D presents the LSV curves of the VMoCoCOx@NF electrode initially and after 10,000 CV cycles, which basically overlap, proving the catalyst has good durability. In addition, to compare the catalytic performances of VMoCoCOx@NF with different mole ratios, the HER polarization curves of VMo_0.5_Co_0.5_COx@NF, VMo_0.6_Co_0.4_COx@NF, VMo_0.4_Co_0.6_COx@NF, and VMo_0.3_Co_0.7_COx@NF were displayed in Figure S9A. Among them, VMo_0.5_Co_0.5_COx@NF needed a small overpotential to reach 10 mA cm^−2^, indicating that its catalytic performance was greatly improved after the introduction of V, Mo, and Co with the optimized mole ratio 1:0.5:0.5. These results confirmed that the three elements incorporated with the optimized mole ratio of 1:0.5:0.5 effectively improved the HER electrocatalytic activity of the catalyst.

### 2.3. Electrocatalytic OER Measurement

**Figure 4:**
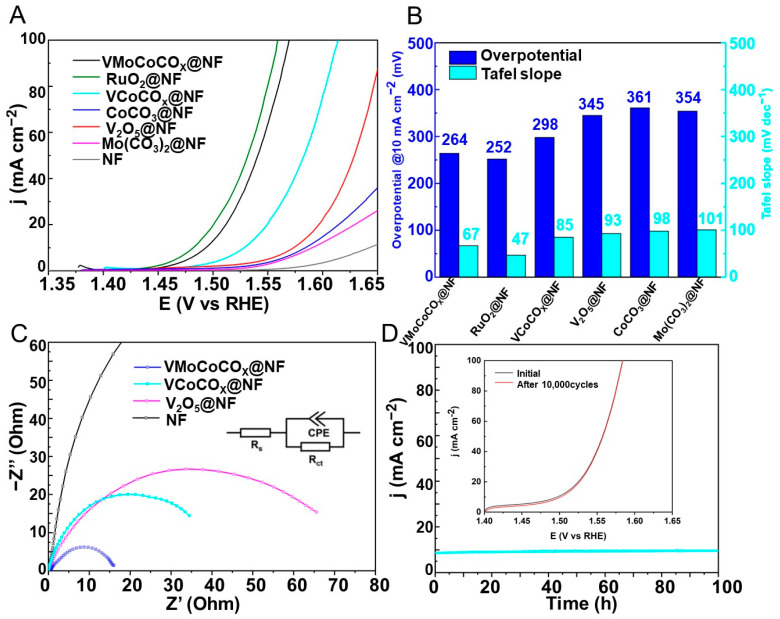
Electrocatalytic properties of the catalysts selected for OER. (**A**) OER LSV polarization curves. (**B**) Comparison of Tafel slopes and overpotentials of the catalysts at a constant *j* = 10 mA cm^−2^. (**C**) EIS curves of the four catalysts. (**D**) Chronoamperometry stability test of VMoCoCOx@NF performed at a constant *j* = 10 mA cm^−2^. The inset showed the LSV curves initially and after 10,000 CV cycles.

Similarly, VMoCoCOx@NF exhibited better OER performance in an O_2_-saturated 1.0 M KOH electrolyte at a current density of 10 mA cm^−2^. To further ascertain the codoping contribution of trimetallic V, Mo, and Co loaded on the bare NF substrate, the OER performance was also assessed using the standard three-electrode system. As depicted in Figure 4A, VMoCoCOx@NF revealed the best OER performance with the lowest overpotential. In Figure 4B and Figure S7B, the overpotential of VMoCoCOx@NF only reached 264 mV, which was far less than those for VCoCO_3_@NF (298 mV), V_2_O_5_@NF (345 mV), CoCO_3_@NF (361 mV), and Mo(CO_3_)_2_@NF (354 mV), and close to RuO_2_@NF (252 mV). The Tafel slope value obtained for VMoCoCOx@NF (67 mV dec^−2^) was also much lower than those of VCoCO_3_@NF (85 mV dec^−1^), V_2_O_5_@NF (93 mV dec^−1^), CoCO_3_@NF (98 mV dec^−1^), and Mo(CO_3_)_2_@NF (101 mV dec^−1^). The lower value of the Tafel slope for VMoCoCOx@NF also reveals the better OER performance of VMoCoCOx@NF, in good agreement with the LSV data. It clearly revealed the superior activity of VMoCoCOx with Ni foam support [25]. The porous NF substrate plays an important role in improving electrocatalytic properties by enlarging the active surface area, accelerating charge/electron transfer, and providing excellent electrical conductivity. The active catalyst loaded on the NF substrate also improved the catalyst–substrate contact for effective charge/electron transfer and rapid release of H_2_/O_2_ during water electrolysis.

Furthermore, as shown in Figure 4C, VMoCoCOx@NF displayed an R_ct_ of 0.66 Ω, which was smaller than those of VCoCOx@NF (19.8 Ω), V_2_O_5_@NF (27.8 Ω), and NF (>60 Ω). VMoCoCOx@NF featured the smallest semicircle diameter with a minimum R_ct_ among the other catalysts, indicating that the dopants reduced the R_ct_, enhanced the electrocatalytic activity, and benefited the OER catalytic kinetics. The V, Mo, and Co elements acted as the exposed active sites for the OER reaction as well. Also, the active catalyst loaded on NF improved catalyst–substrate contact, enabling efficient charge/electron transfer during water electrolysis, thereby showing its better electronic conductivity and faster OER catalytic kinetics [15]. The R_ct_ was also consistent with the Tafel slopes and overpotentials. Based on the above, in situ growth of VMoCoCOx@NF also contributed to a decrease in R_ct_. The trimetallic doping of VMoCoCOx@NF was critical to the enhancement of the OER catalytic kinetics.

The stability of VMoCoCOx@NF during OER was assessed using chronoamperometry (*i*–*t*) and CV tests. As shown in Figure 4D, its chronoamperometric curve displayed that the constant current density of 10 mA cm^−2^ was barely degenerated after the 100 h test. The inset in Figure 4D depicts that the LSV curves of the as-prepared electrode almost overlap with those of the initial and after 100 h tests, which proves the sample has outstanding stability. The aforementioned comparative study showed that the VMoCoCOx@NF had a smaller overpotential and Tafel slope than those of the compared catalysts, thus confirming its superior OER performance (Table S8). Moreover, as shown in Figure S9B, VMo_0.5_Co_0.5_COx@NF manifested better OER activity with a smaller overpotential, which was clearly less than those of VMo_0.6_Co_0.4_COx@NF, VMo_0.4_Co_0.6_COx@NF, and VMo_0.3_Co_0.7_COx@NF, implying a significantly enhanced electrocatalytic performance towards OER after the incorporation of trimetallic V, Mo, and Co with an optimized mole ratio of 1:0.5:0.5. The optimized experimental results were also consistent with the RSM simulation.

### 2.4. Electrocatalytic Performance for Overall Water Splitting

Electrocatalytic water splitting refers to the process by which water molecules are cleaved into H_2_ and O_2_ by catalyzing the separation of H_2_O through the catalyst electrodes in an electrolytic cell (2H_2_O → 2H_2_↑ + O_2_↑). Considering the excellent OER and HER activities of VMoCoCOx@NF, by using the as-prepared catalyst serving as both anode (4OH^−^ → 2H_2_O + O_2_↑ + 4e^−^) and cathode (2H_2_O + 2e^−^→ 2OH^−^+ H_2_↑) for overall water splitting, a symmetric two-electrode water electrolyzer was constructed in a 1.0 M KOH electrolyte.

To investigate the influence of VMoCoCOx@NF with different V/Mo/Co mole ratios on the alkaline HER and OER, the mole ratios of metal precursors were optimized by RSM. Herein, single-factor experiments confirmed that the mole amounts of Mo and Co were sensitive factors for the catalyst formulation, and these sensitive factors were optimized by RSM. The VMo_0.6_Co_0.4_COx@NF, VMo_0.5_Co_0.5_COx@NF, VMo_0.4_Co_0_._6_COx@NF, and VMo_0.3_Co_0.7_COx@NF were selected as target electrocatalysts (Table S1). When V was set to 1 mole, Mo mole (χ_1_) and Co mole (χ_2_) were selected as independent variables (Table S2). Mo/Co mole ratio, O_2,_ and H_2_ production were chosen as responses. Figures S1 and S2 present the single and combining effects of independent variables on all responses. The 3D response surface contour plots (3D-RSCP) were generated accordingly. The adequacy of the model was rigorously evaluated by analysis of variance (ANOVA) with the corresponding results listed in Tables S4 and S5. The coefficients of determination R-squared (R^2^), adjusted R^2^ (adj. R^2^), and predicated R^2^ (pred. R^2^) were used to evaluate the fitting model. The difference between adj. R^2^ and pred. R^2^ was less than 0.2, which indicated that the model fitted all data (Figure S2 and Table S3 for details).

**Figure 5:**
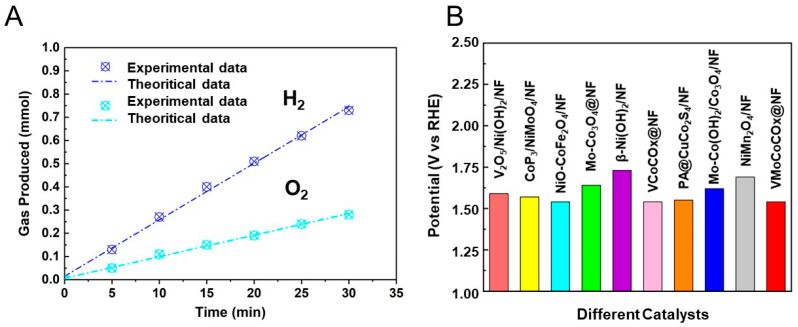
(**A**) The overall water splitting activity of VMoCoCOx@NF in the alkaline electrolyzer. The experimentally measured and theoretically calculated millimolars of H_2_ and O_2_ as a function of time were plotted at 5 min intervals during the water splitting at a constant *j* = 10 mA cm^−2^. (**B**) The comparison of potentials between the VMoCoCOx@NF electrolyzer and other reported NF-supported transition metal-based catalysts [17,22,26,27,28,29,30,31,32].

The generated volumes of H_2_ and O_2_ were the response values of optimization with help from RSM. In Table S1, the VMoCoCOx@NF fabricated with NH_4_VO_3_: (NH_4_)_2_MoO_4_:CoCl_2_ mole ratio of 1:0.5:0.5 showed maximum yields of H_2_ (96.3%) and O_2_ (92.5%), which exhibited excellent catalytic activity and should be a most promising bifunctional catalyst. Figure S2 illustrates a good match between predicted and actual generated volumes of O_2_ and H_2_. The predicted yields of H_2_ (94.0%) and O_2_ (90.2%) were similar to the actual yields of H_2_ (96.0%) and O_2_ (91.6%) (Table S6). The comparison points were evenly distributed on both sides of the fitted line. On the other hand, LSV measurement is one of the most common methods for rapid assessment of electrocatalyst activity. The LSV polarization curves of the optimized VMo_0.5_Co_0.5_COx@NF for both HER and OER showed lower overpotentials than the other catalysts, as depicted in Figure S9. Therefore, Equations (S1) and (S2) in the SI provide a good fit to the data and can be used to predict the uninvestigated experimental variables. The prediction model after the ANOVA on the responses predicted the optimal V:Mo:Co mole ratio of 1.0:0.538:0.483. The VMoCoCOx@NF with an optimal mole ratio of 1:0.5:0.5 revealed outstanding bifunctional electrocatalytic activity for overall water splitting by achieving 10 and 100 mA cm^−2^ at cell voltages of 1.54 V and 1.61 V in 1.0 M KOH electrolyte. Inspired by the superb OER and HER performances of VMoCoCOx@NF in alkaline media, the catalyst served as both anode and cathode by applying electric voltage to an alkaline electrolyzer. Numerous tiny bubbles of H_2_ and O_2_ were discharged from the electrode surface. Moreover, to investigate the electrocatalytic efficiency of the VMoCoCOx@NF||VMoCoCOx@NF electrolyzer, the generated H_2_ and O_2_ gases were monitored using gas chromatography (GC). The millimoles of O_2_ and H_2_ generated from the water splitting at 5 min intervals are intuitively observed in Figure 5A. As expected, after 30 min of water electrolysis, 0.298 millimoles of O_2_ and 0.694 millimoles of H_2_ were collected at the anode and the cathode, respectively. Concomitantly, the evolution millimolar ratio of the generated H_2_ and O_2_ bubbles was close to 2:1, which was consistent with the theoretically calculated value produced by water electrolysis. The agreement between experimental and theoretical values indicated the gas-generated efficiencies were 96.3% for H_2_ and 92.5% for O_2_, respectively, which were closer to 100%, proving that VMoCoCOx@NF was a promising bifunctional catalyst (Table S1). The contribution of the NF-supported VMoCoCOx@NF catalyst was also verified. Table S8 summarizes and compares the electrocatalytic activity (HER, OER, and overall water splitting) of the VMoCoCOx@NF catalyst with those of recently reported NF-supported transition metallic electrocatalysts in 0.1 M KOH electrolyte, from which it is found that the HER/OER results as well as the water-splitting results of VMoCoCOx@NF are better than those of the recently reported bifunctional electrocatalysts [17,22,26,27,28,29,30,31,32]. The overall water-splitting experiment was performed in a two-electrode electrolyzer consisting of a VMoCoCOx@NF catalyst as both the positive and negative electrodes. Notably, the water-splitting penitential of the VMoCoCOx@NF||VMoCoCOx@NF alkaline water electrolyzer was lower than that of the others, as displayed in Figure 5B. It needs a voltage of 1.54 V to afford a water-splitting current density of 10 mA cm^−2^ with tiny bubbles of H_2_ and O_2_ evolution on both electrodes. The developed catalyst had a promising application in place of the noble metal electrocatalyst, which might be related to the following factors [33,34]. First, the clustered VMoCoCOx nanosheet structure was verified by FESEM analysis. The catalyst could expose more active sites, thereby accelerating water electrolysis. Second, the multimetallic active sites facilitate the bifunctional electrocatalytic behavior due to heteroatom doping of V/Mo/Co. Third, discontinuous crystal fringes enlarged the surface area on VMoCoCOx@NF, favoring the adsorption, activation, and dissociation of water molecules. Fourth, porous NF structure also greatly facilitates the generation and diffusion of H_2_ and O_2_ bubbles and effectively enhances water electrolytic efficiency. Finally, the integrated catalytic material design reduces the charge/electron transfer resistance during the water electrolysis. Herein, a novel electrocatalyst with V and Mo codoping for water splitting was first developed. The excellent electrocatalytic performance of VMoCoCOx@NF was assigned to the synergistic effect of 2p, 3d, and 4d vacancies and multimetallic codoping, including increased valence states and enhanced catalytic kinetics. The electronic structure benefits from the assembled forest-like nanosheet architecture with more active sites, a short charge/electron transfer path, and easy infiltration of electrolytes [35].

**Figure 6:**
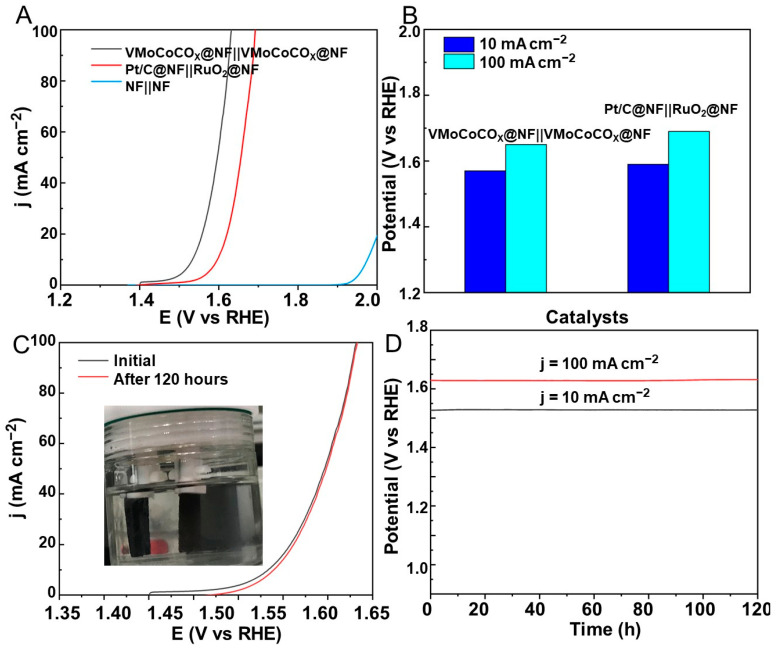
(**A**) The polarization curves of VMoCoCOx@NF||VMoCoCOx@NF, Pt/C@NF||RuO_2_@NF and NF||NF-coupled catalysts in a two-electrode configuration. (**B**) Comparison of the overpotentials between VMoCoCOx@NF||VMoCoCOx@NF and RuO_2_@NF||Pt/C@NF at *j* = 10 mA cm^−2^ and 100 mA cm^−2^. (**C**) The polarization curves before and after the 120 h stability test. Inset (**C**) showed the VMoCoCOx@NF||VMoCoCOx@NF alkaline water electrolyzer. (**D**) Chronopotentiometry stability tests performed at constant *j* = 10 mA cm^−2^ and 100 mA cm^−2^ for 120 h.

Figure 6A displays the LSV curves of VMoCoCOx@NF||VMoCoCOx@NF, Pt/C@NF||RuO_2_@NF, and NF||NF electrolyzers. The VMoCoCOx@NF||VMoCoCOx@NF alkaline water electrolyzer required a cell voltage of 1.54 V to achieve a current density of 10 mA cm^−2^, which was lower than those of the commercial Pt/C@NF||RuO_2_@NF (1.56 V) and NF||NF (1.96 V) catalysts. The better performance outperformed most previously reported catalysts (Table S8) [5]. More significantly, the catalytic activity of VMoCoCOx@NF was increased when V and Mo were codoped into CoCOx, demonstrating that the synergetic effect of V and Mo was mainly responsible for improving the bifunctional performance. Figure 6B shows the VMoCoCOx@NF||VMoCoCOx@NF alkaline water electrolyzer operating at current densities of 10 and 100 mA cm^−2^, the voltages of 1.54 V and 1.61 V, respectively, which are superior to Pt/C@NF||RuO_2_@NF electrolyzer (voltages of 1.56 V and 1.67 V, respectively). Therefore, the VMoCoCOx@NF catalyst possesses promising applications for water electrolysis in reality.

The durability of the electrode is a key parameter to evaluate the performance of an electrocatalyst. The catalyst was subjected to continuous CV for durability testing, and the polarization curves were plotted after 10,000 CV cycles. Figure 6C shows almost similar polarization curves before and after the 120 h test, which is conducive to the long-term stable use of the catalyst. The inset in Figure 6C shows the overall water electrolytic cell device using VMoCoCOx@NF as both electrodes. In addition to activity, long-term durability is another critical aspect of catalysts, especially for nanostructural catalysts. Figure 3D and Figure 4D show the superior stabilities for HER and OER at a constant current density of 10 mA cm^−2^. Indeed, the stability measured at a constant current density is more reliable. Considering the large mass loading on the NF substrate, the cyclic stability of over-water splitting should be measured at a large current density. In Figure 6D, when constant current densities of 10 mA cm^−2^ and 100 mA cm^−2^ were applied for overall water splitting, the *v*–*t* curves of the fabricated VMoCoCOx@NF required cell voltages of 1.54 V and 1.61 V. The potentials exhibited negligible changes over 120 h tests with robust stability. As displayed in Figure S10, there were no obvious changes in the structure (XRD), morphology (SEM), or chemical state (XPS) of the sample after stability testing, indicating that the catalyst had very good performance.

Interestingly, as shown in Figure 3B and Figure 4B, the VMoCoCOx@NF catalyst exhibited the highest HER and OER activities among the synthesized samples, except for the commercial Pt/C and RuO_2_. Nevertheless, the overall water splitting experiments were performed in a two-electrode configuration composed of VMoCoCOx@NF catalyst as anode and cathode. Figure 6A,B displays that the water-splitting potential of the VMoCoCOx@NF||VMoCoCOx@NF cell (1.54 V) is lower than that of the Pt/C@NF||RuO_2_@NF cell (1.59 V). This fact can be explained by the high over-water-splitting activity of VMoCoCOx@NF||VMoCoCOx@NF cell.

### 2.5. DFT Theoretical Calculation

**Figure 7:**
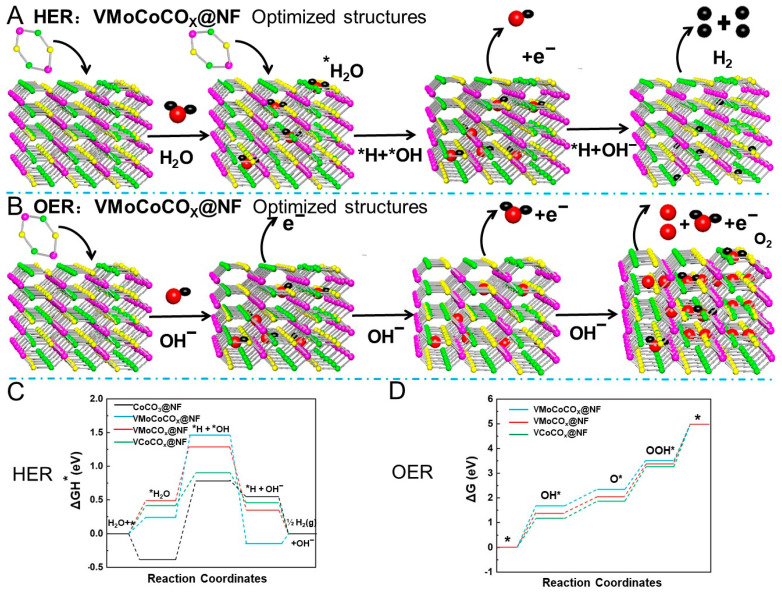
DFT calculation. (**A**) DFT-optimized structures of OH*, H*, and H_2_O* intermediates on the Co and Mo sites of VCMoCoCOx for HER. (**B**) DFT-optimized structures of *, OH*, O*, and OOH* intermediates on the Co and Mo sites of VCMoCoCOx for OER. Color code: The Co atom is green, the Mo atom is purple, the V atom is yellow, the O atom is red, and the H atom is black. (**C**) ΔG_H*_ for the HER mechanism on the surface of CoCO_3_@NF, VMoCoCOx@NF, VMoCOx@NF, and VCoCOx@NF. (**D**) ΔG for the OER mechanism on the surfaces of VMoCO_x_@NF, VMoCoCOx@NF, and VCoCOx@NF. The dotted lines in (**C**,**D**) represent the ΔG jumps at each step.

The DFT theoretical calculation in this work was theoretically performed to elucidate the origin of the improved electrocatalytic performance of VMoCoCOx@NF in alkaline media. In order to understand its contribution to the electrocatalytic activity, in the computation hydrogenation electrode (CHE) modeling, a VMoCoCOx@NF surface slab with six-atomic hexagonal clustered nanosheets was first constructed to study the reaction intermediate steps involved in both HER and OER. As revealed in Figure 7A, the optimized geometry structures of OH*, H*, and H_2_O* intermediates on the active sites of VMoCoCOx were observed for HER. Fortunately, the H* spontaneously moved between the interfacial V and Mo, suggesting that the doping metallic atoms could provide active sites. Meanwhile, the structural features and the Gibbs free energy (ΔG) profiles of VMoCoCOx@NF were verified by constructing the correlative theoretical model. The alkaline catalytic process proceeds through either the Volmer–Heyrovrosky or Volmer–Tafel reactions (SI). The alkaline HER mechanism of VMoCoCOx@NF was proposed through the DFT calculation: VMoCoCOx@NF served to favor the adsorption, activation, and dissociation of H_2_O (* + H_2_O → *H_2_O → H* + *OH) to produce H* and *OH. The clustered VMoCoCOx nanosheets provided the active sites for H_2_ generation (*OH + e^−^ → OH^−^; H* + H*→ H_2_↑). To examine the H* adsorption/H_2_ desorption step (the Heyrovsky step), the ΔG_H*_ values for all possible active sites were calculated [36]. Both pathways involve the adsorption of H_2_O, electrochemical reduction into adsorbed H atoms and OH^−^, and desorption of H_2_.

In an alkaline environment, OER has occurred on the anode. During the adsorption process, the velocity of the adsorbent decreases slowly; it finally stops on the surface slab of the catalyst, and part of the energy is released, which is the adsorption energy. The more negative the adsorption energy, the faster the charge/electron transfer from active sites to H_2_O molecules. The adsorption energy is the critical factor for validating these catalysts’ theoretical activity [20,22]. The V, Mo, and Co atoms of VMoCoCOx@NF are the exact active sites for OER. The adsorption energies at the active sites of V, Mo, and Co of VMoCoCOx@NF are −6.88 eV, −7.39 eV, and −5.43 eV, respectively. In this case, the geometric structures of OER intermediate adsorption on VMoCoCOx@NF are depicted in Figure 7B. The OER catalytic activity can follow four steps: (1)–(4).

M* + OH^−^→M-OH* + e^−^  [OH^−^ first adsorption step (1)]

M-OH* + OH^−^ →M-O* + H_2_O + e^−^  [O-H bond breaking step (2)]

M-O* + OH^−^ →M-OOH* + e^−^  [OH^−^ second adsorption step (3)]

M-OOH* + OH^−^ →M* + O_2_↑ + H_2_O + e^−^  [oxygen forming step (4)]

where M is the active site of V, Mo, and Co elements. First, OH^−^ in the electrolyte is adsorbed by the catalytically excited active sites (M*) to produce M-OH*. Second, the produced M-OH* reacts with OH^−^ to form M-O*, and the produced M-O* further reacts with OH^−^ to produce intermediate M-OOH*. Finally, the intermediate M-OOH* reacts with OH^−^ to generate O_2_ [37].

For the HER, Figure 7C displays the reaction intermediate steps and corresponding ΔG_H*_ values involved in the HER process on the catalyst, which unveils the origination of electrocatalytic HER activity. The RDS for H_2_ generation was the conversion of H* to H_2_, which was the Tafel step. These profiles compared the four computed ΔG_H_* values for different possible adsorption sites of CoCOx@NF, VMoCOx@NF, VMoCoCOx@NF, and VCoCOx@NF, respectively. According to the ΔG_H_* values of HER on the as-prepared catalysts, RDS was the step involved in the formation of H*. The ΔG_H*_ of RDS were −0.04 eV, 0.07, 0.23, and 0.37 eV for the HER activities of VMoCoCOx@NF, VCoCOx@NF, VMoCO_3_@NF, and CoCO_3_@NF, respectively. Similarly, V doping of CoCO_3_@NF significantly reduced the ΔG_H*_ from 0.37 eV (CoCO_3_@NF) to 0.07 eV (VCoCOx@NF). The lower the ΔG_H*_ is, the higher the HER activity. The dissociated H* were adsorbed by the V and Mo atoms on the surface of the VMoCoCOx@NF electrode. The ΔG_H*_ of VMoCoCOx@NF for water dissociation to generate H_2_ was −0.04 eV, which was close to the optimum value ΔG_H*_ = 0 for an ideal HER electrocatalyst, and the H_2_O could be effectively activated on its surface. The above results suggested that the ΔG_H*_ value of the VMoCoCOx@NF catalyst was lower than that of the VCoCOx@NF, VMoCO_3_@NF, and CoCO_3_@NF catalysts. The significantly decreased ΔG_H*_ value demonstrated that the heterointerface effect between VMoCoCOx and NF also speeded up the H_2_ release on the developed catalyst, which further promoted HER activity in alkaline media. The DFT results revealed that the VMoCoCOx@NF exhibited excellent water-splitting capability. The structure of VMoCoCOx@NF was moderated by the dopants of V and Mo, reducing its adsorption of H_2_O and increasing the possibility of H_2_O dissociation. The theoretical calculation findings are basically consistent with the experimental data.

However, a theoretical study depicted the OER process in alkaline electrolytes. Water molecules underwent a complex four-step electron “cross-mountain” process (* → *OH → *O → *OOH → * + O_2_). The OER activity was related to the corresponding ΔG of *, *OH, *O, and *OOH intermediates. The total ΔG barrier of O_2_ generated from water electrolysis was 4.92 eV. In theoretical conditions, the ΔG value for each step of water electrolysis was 1.23 eV, but the actual OER process was usually complex, and the ΔG was different for each step. Actually, the alkaline OER process (4OH^−^ → O_2_↑ + 2H_2_O + 4e^−^) can be described by the four-step water associative mechanism [4].

The OER activity of the catalyst was significantly related to the characteristics of the surface metal sites. For the DFT calculations, the adopted active sites were V, Mo, and Co on VMoCoCOx@NF. In Figure 7D, the affinity of VMoCoCOx@NF with *O in water was slightly higher; step (3) was the RDS. The discussion concerning the OER process showed the RDS was found to be deprotonation of absorbed *O, which was in good accordance with Ding’s finding [36]. The decreased ΔG of the RDS suggested a higher intrinsic OER activity.

In Figure 7D, the ΔG values of different steps for *, OH*, O*, and OOH* were calculated to reveal the origin of OER activity. The star * is the active site. The theoretical overpotential of OER on VMoCoCOx@NF was determined by the following equation:

η_OER_ = {max[ΔG_1_, ΔG_2_, ΔG_3_, ΔG_4_]/e} − 1.23 [V]

where ΔGn (n = 1–4) is the ΔG along the OER process (4.92 eV/4 = 1.23 eV). DFT calculation indicated that VMoCoCOx@NF processed a smaller ΔG than those of VMoCO_x_@NF and VCoCOx@NF for PDS in the four-step OER reactions, confirming the positive effect of VMoCoCOx@NF on catalyzing OER.

The ΔG diagrams of VMoCOx@NF, VMoCoCOx@NF, and VCoCOx@NF calculated for the OER are shown in Figure 7D. What is more, the value of ΔG_O*_ − ΔG_OH*_ is often chosen as the OER descriptor [4]. Herein, the RDS was the deprotonation step (HO* → O*+ H^+^ + e^−^) with ΔG_2_^0^ of 0.60 eV, 0.62 eV, and 0.64 eV for VMoCoCOx@NF (1.97 eV − 1.37 eV = 0.60 eV), VMoCOx@NF (2.27 − 1.65 = 0.62 eV), and VCoCOx@NF (1.82 − 1.18 = 0.64 eV), respectively. Also, as Figure 7D suggested, the *OH-M intermediate was thermodynamically slightly more stable than *OH-M because of its lower ΔG_2_^0^. Therefore, the affinity of VMoCoCOx@NF with *OH in water was also slightly higher, implying higher surface wettability, which was further verified by the HRTEM test (0.190 nM, 0.285 nM). The codoped V and Mo almost directly took part in the OER procedure. According to DFT computations, the third step is the key process. The electronic modulation from co-doping heteroatoms into composites enhances the electrical conductivity owing to the synergistic effect among V_2_O_5_, MoO_3_, and/or CoCO_3_ that accelerates charge/electron transfer and provides more additional active sites.

The presence of V, Mo, and Co elements substantiated the trimetallic doping of VMoCoCOx@NF, which synergistically improved the active sites of the synthesized hybrid catalysts. For the OER and HER activities, the V, Mo, and Co ions in VMoCoCOx@NF were considered active sites among V_2_O_5_, MoO_3_, and/or CoCO_3_ for each reaction. The construction of multiple heterointerfaces in the VMoCoCOx@NF hybrid catalyst can synergistically improve the charge/electron transfer kinetics and expose more active sites for overall water splitting.

The factors leading to the good bifunctional HER/OER activity of the fabricated catalyst are to facilitate charge-transfer kinetics and enrich more electrically connected active sites. Herein, V, Mo, and Co dopants synergistically enrich more additional active sites on VMoCoCOx@NF, and NF acts as a conducting substrate. The advantage of this heterostructure is conveyed by its fabrication with enriched active sites acting as bridged sites from dopants, which facilitates charge-transfer behavior and ameliorates the catalytic activity of the catalyst for overall water splitting.

## 3. Experimental

Additional experimental details for “Chemicals and Materials” and “Density Functional Theory (DFT)” were provided in the SI.

### 3.1. Preparation of Electrocatalysts

Scheme 1 illustrates the fabrication procedures of VMoCoCOx@NF nanoarrays [17]. The Mo and V-codoped CoCOx nanosheets loaded on the NF substrate were designed to be fabricated as a highly efficient bifunctional catalyst by facile hydrothermal deposition. Prior to the preparation, a piece of NF with the size of 3 × 1 × 0.1 cm^3^ was treated with 1.0 M HCl to remove surface impurities by ultrasonication for 15 min, washing with deionized (DI) water and ethanol consecutively for 10 min, followed by drying in a vacuum oven for 8 h. Specifically, 15.0 mL DI water, 0.18 g NH_4_VO_3_, 0.15 g (NH_4_)_2_MoO_4_, 0.18 g CoCl_2_⋅6H_2_O, 0.90 g NaOH, and 1.00 g CH_4_N_2_O were successively added to a 100 mL beaker. Afterward, the treated NF was immersed in the beaker. Subsequently, the mixed solution was ultrasonicated for 5 min, followed by adding 1.00 g of (NH_4_)_2_S_2_O_8_. After stirring the solution at 25 °C for 12 h, the above solution with NF was transferred to a 25 mL Teflon-lined stainless-steel autoclave and maintained at 150 °C for 10 h. Finally, the as-prepared catalyst was washed with DI water and ethanol in sequence and dried in a vacuum oven at 60 °C for 10 h. Two control samples of VCoCOx@NF and VMoCOx@NF were also fabricated under the same conditions for comparison.

### 3.2. Characterization

The morphology, structure, and elemental information of the prepared catalysts were measured by FESEM-EDX (JEOL JSM-7600F, JEOL, Tokyo, Japan, operating at 5 kV). The crystalline/amorphous phase and lattice spacing were identified using HRTEM (JEM-2100, JEOL, Tokyo, Japan, operating at 200 kV). HRTEM simulation was performed using JEMS 2014 software. The crystallographic identification was acquired by XRD (Ultima IV, Rigaku, Tokyo, Japan, Cu Kα radiation, λ = 1.5406 Å). The test conditions were 40 kV, 40 mA, and a scan rate of 1 × 10^−2^ degree/second in the 2θ range from 10° to 80°. The surface elemental composition and valence states were investigated by XPS (PHI 5000 VersaProbe, ULVaC-Phi, Kanagawa, Japan, Al Kα radiation, *hν* = 1486.6 eV), and data were fitted by the XPSPEAK 41 software. FTIR analysis was conducted on an FTIR spectrometer (VERTEX 80V, Bruker, Ettlingen, Germany) from 450 to 4000 cm^−1^ at 0.5 cm^−1^ spectral resolution. An inductively coupled plasma–optical emission spectrometer (ICP-OES) (Avio 220, PerkinElmer, Waltham, MA, USA, operating under 15.0 L min^−1^ coolant Ar gas, 0.9 L min^−1^ plasma Ar gas, and 0.9 L min^−1^ nebulizer Ar gas) was used to detect the element molar ratios.

### 3.3. Electrochemical Measurements

Electrochemical measurements were conducted on an electrochemical workstation (CHI 760E, Chenhua Instrument Company, Shanghai, China) connected to a conventional three-electrode system in 1.0 M KOH electrolyte at ambient temperature (25 °C). The fabricated VMoCoCOx@NF catalyst was tailored as 1 × 1 cm^2^ for direct use as the working electrode. A graphite plate and normal calomel (Hg/HgO) were used as the counter electrode and reference electrode, respectively. The measured potentials (E_Hg/HgO_) were calibrated to reversible hydrogen electrode potentials (E_RHE_) according to the Nernst equation: E_RHE_ = E_Hg/HgO_ + 0.098 V + 0.059 × pH. All data presented were corrected for ohmic resistance. The HER/OER activities and Tafel slopes of the tested catalysts were compared, respectively. The HER and OER polarization tests were performed in N_2_-saturated and O_2_-saturated electrolytes, respectively. The LSV polarization curves of both OER and HER were recorded at a scan rate of 5 mV s^−1^. The EIS was measured from 100 kHz to 0.01 Hz with an amplitude of 5 mV. The cycling stability of VMoCoCOx@NF was measured at a scan rate of 50 mV s^−1^ by CV for 10,000 CV cycles. The acquisition of the Tafel equation, *η* = a + b log|*j*|, is based on the LSV calculation, where b is the Tafel slope and *j* is the current density. The ECSA of the electrode was evaluated using the electrochemical C_dl_ and the specific capacitance (C_s_) (ECSA = C_dl_/C_s_). Cs is 0.04 mF cm^−2^ for transition metal-based materials in alkaline media [35]. C_dl_ was measured by a CV test under scan rates ranging from 10 to 120 mV s^−1^. The two-electrode configuration was used to perform overall water splitting. The permanency of the optimized VMoCoCOx@NF catalyst toward HER, OER, and overall water splitting was evaluated using the chronoamperometry (*j*–*t*) or chronopotentiometry (*v*–*t*) methods. The generated volumes of H_2_ and O_2_ gas were measured using GC (7890A, Agilent Technologies, Santa Clara, CA, USA) (GB/T 28124-2011) [38]. Besides VMoCoCOx@NF, VCoCOx, VMoCOx, CoCO_3_, V_2_O_5_, commercial Pt/C, and RuO_2_ loaded on NF were also used as working electrodes for comparison.

### 3.4. Density Functional Theory (DFT) Calculations

All spin-polarized DFT calculations were completed using the Vienna Ab initio software package (VASP5.4.4) [35]. The projector augmented wave (PAW) method was used to describe the electron–ion interaction, with 1s of H, 2s2p of C and O, 3d4s of V and Co, and 4d5s of Mo considered valence electrons. The electron–electron exchange-correlation functions were calculated by Perdew–Burke–Ernzerhof (PBE) in the generalized gradient approximation (GGA) for structure and energy optimization [39]. For an accurate description of V (3d), Co (3d), and Mo (4d) electrons, Hubbard effective terms U_eff_ (Co) = 3.32 eV, U_eff_ (Mo) = 4.38 eV, and U_eff_ (V) = 3.4 eV were added to the PBE functional via the rotationally invariant approach. The U_eff_ values were obtained from the Materials Project database [40]. For all periodic structures, the *k* point of the Brillouin zone based on monkhorst-pack grid mesh was 2 × 1 × 1, the truncation energy was 500 eV, and the convergence criteria of force and energy were 10^−2^ eV Å^−1^ and 10^−5^ eV, respectively [41].

However, the VMoCoCOx (112) plane reproduces the “distorted hexagonal clustered nanosheets” structure very well. The optimized bulk face-centered cubic V, Mo, and Co structure has lattice constants of 3.030 Å, 3.147 Å, and 2.507 Å, which are much closer to their experimental lattice constants (3.046 Å, 3.123 Å, and 2.531 Å). The lattice constants for optimized bulk hexagonal VMoCoCOx are a = 4.577 Å and c = 4.211 Å (experimental values: a = 4.562 Å and c = 4.127 Å). The optimized bulk structure was used to construct the surface slab model [42]. For VMoCoCOx@NF, a V- and Mo-terminated surface (112) slab of 3 layers was constructed. The VMoCoCOx hexagonal clustered nanosheets loaded on the top surface of the NF substrate (111), with V and Mo terminated, interact with the NF (111) surface to make an effective catalytic interface. Therefore, the VMoCoCOx nanosheets in the hybrid model experienced compressive strain from the NF substrate. The two sides of the clustered hexagonal nanosheets correspond to the V- and Mo-terminated surfaces of VMoCoCOx (112). In Figure S11A, the structural characteristics for constructing VMoCoCOx@NF were explored by employing the methods of model chemistry, including ab initio computation and DFT calculation. As illustrated in the VMoCoCOx@NF structure diagram, except for V, Mo, and Co, the CO_3_^2−^ anions composed of C and O should intersperse in the structure constructed by V, Mo, and Co.

In the H*_Slab_ model, the adsorption energy of hydrogen (ΔE_H*_) was expressed as ΔE_H*_ = E_H*slab_ − (E_slab_ + 0.5E_H2_). Meanwhile, a catalyst with ΔG = 0, or 1.23 eV, is an excellent candidate for OER. The ΔG correlations were calculated by zero-point energy and entropic correction [43]. The climbing image nudged elastic band (CI-NEB) and the dimer approach were used to simulate the reaction pathway.

## 4. Conclusions

In this work, the molybdenum and vanadium codoped cobalt carbonate nanosheets loaded on nickel foam (VMoCoCOx@NF) were fabricated, and the mole ratio of V/Mo/Co was optimized by response surface methodology. The optimized VMoCoCOx@NF catalyst displays superior HER and OER performance with lower overpotentials and smaller Tafel slopes, along with robust stability in the 1.0 M KOH electrolyte, which is associated with the synergy of V_2_O_5_, MoO_3,_ and CoCO_3_ and heterostructural features. Also, an alkaline water electrolyzer with VMoCoCOx@NF as both the cathode and anode enables current densities of 10 and 100 mA cm^−2^ with voltages of 1.54 V and 1.61 V, respectively. Through detailed investigation, the working mechanism of the yielded advanced HER and OER activity was clarified via the DFT simulations. More importantly, this work offers an excellent and highly cost-effective self-supported transition metal-based bifunctional catalyst material for practical use in clean energy H_2_ generation.

## Data Availability

No new data were created except for the data in the Supplementary Materials and the paper.

## Supplementary Materials

The following supporting information can be downloaded at: https://www.mdpi.com/article/10.3390/molecules29153591/s1. Optimize mole ratios of the fabricated electrocatalysts with response surface methodology (RSM); Detailed DFT calculations; Figure S1: 3-D response surface plots and contour plots for the as-prepared catalysts with different mole ratios of Mo and Co; Figure S2: Fit plots for predicted and actual values; Figure S3: XRD patterns of VMoCoCOx@NF, VCoCOx@NF, CoCOx@NF and V_2_O_5_@NF on the same coordinates; Figure S4: (A) HRTEM image of VMoCoCOx@NF. (B) The lattice spacing distributions corresponding to 0.261 nm for the (310) plane and (C) 0.190 nm for the (112) plane; Figure S5: The FTIR spectra of VMoCoCOx@NF, VCoCOx@NF and V_2_O_5_@NF; Figure S6: Nyquist plots of HER for the four catalytic electrodes; Figure S7: Comparison of Tafel slopes of the catalysts selected for HER (A) at a constant *j* = −10 mA cm^−2^ and OER (B) at a constant *j* = 10 mA cm^−2^; Figure S8: The original CV curves of VMoCoCOx@NF (A), VCoCOx@NF (B) and V_2_O_5_@NF (C) recorded at various scan rates in the non-faradaic region for the estimation of C_dl_. (D) Estimation of C_dl_ values of VMoCoCOx@NF, VCoCOx@NF, CoCOx@NF and V_2_O_5_@NF by plotting the ratios of catalyst Δ*J* to scan rate.; Figure S9: The polarization curves of VMo_0.5_Co_0.5_COx@NF, VMo_0.6_Co_0.4_COx@NF, VMo_0.4_Co_0.6_COx@NF, and VMo_0.3_Co_0.7_COx@NF for HER (A) and OER (B); Figure S10: The structure (XRD)(A), morphology (SEM)(B) and chemical state (XPS)(C) of the sample before and after stability testing; Figure S11: (A) The crystal plane of VMoCoCOx (112), (B) Crystal structure of NF (111) in top view. Table S1. Optimized the catalysts with different mole ratios of V, Mo and Co based on the experimental production of O_2_ and H_2._ Table S2. Levels of independent variables for the response surface design for VMoCoCOx@NF with different mole ratios of Mo and Co. Table S3. Response surface optimal (custom) design and the experimental and predicted production of O_2_ and H_2_ by VMoCoCOx@NF with different ratios of Mo and Co. Table S4. ANOVA for response surface quadratic model for O_2_ production of VMoCoCOx@NF with different ratios of Mo and Co. Table S5. ANOVA for response surface quadratic model for H_2_ production of VMoCoCOx@NF with different ratios of Mo and Co. Table S6. Optimum values for the ratio of Mo and Co for VMoCoCOx@NF and the production of O_2_ and H_2._ Table S7. XRD data of the target materials. Table S8. Comparison of the recently reported NF-supported transition metallic electrocatalysts for the electrocatalytic activities (HER, OER, and overall water splitting) in 0.1 M KOH electrolyte.
