# Supplementary material for: Molybdenum and Vanadium-Codoped Cobalt Carbonate Nanosheets Deposited on Nickel Foam as a High-Efficient Bifunctional Catalyst for Overall Alkaline Water Splitting
Source: Molecules. 2024 Jul 30;29(15):3591. doi: 10.3390/molecules29153591 (PMC11314115; doi:10.3390/molecules29153591)
Supplement: Supplementary file 1 [file molecules-29-03591-s001.zip › molecules-3120932-supplementary.pdf]

Supporting information (SI)

Molybdenum and Vanadium-Codoped Cobalt Carbonate Nanosheets Deposited on Nickel Foam as a High-Efficient Bifunctional Catalyst for Overall Alkaline Water Splitting

Wenxin Wang [1,2], Lulu Xu [2,3], Ruilong Ye [2,3], Peng Yang [2,3], Junjie Zhu [1,2], Liping Jiang [1,2,*] and Xingcai Wu [2,3,*]

[1] State Key Laboratory of Analytical Chemistry for Life Science, Nanjing University, Nanjing 210023, China
[2] School of Chemistry and Chemical Engineering, Nanjing University, Nanjing 210023, China
[3] Key Laboratory of Mesoscopic Chemistry of MOE, Nanjing University, Nanjing 210023, China
* Correspondence: jianglp@nju.edu.cn (L.J.); wuxingca@nju.edu.cn (X.W.)

Contents

1. Optimize Mole Ratios of the Fabricated Electrocatalysts with Response Surface Methodology (RSM)

2. Additional Experimental Details

3. Experimental Figures and Tables

1. Optimize Mole Ratios of Electrocatalysts with Response Surface Methodology (RSM)

Table S1. Optimized the catalysts with different mole ratios of V, Mo and Co based on the experimental production of O_2 and H_2.

	V/Mo/Co	O_2(ml)	%	H_2	%
$VMo_{0.6}Co_{0.4}COx$@NF	1.0/0.6/0.4	3.5(4.0)	87.5%	6.8(8.0)	85.0%
$VMo_{0.5}Co_{0.5}COx$@NF	1.0/0.5/05	3.7(4.0)	92.5%	7.7(8.0)	96.3%
$VMo_{0.4}Co_{0.6}COx$@NF	1.0/0.4/0.6	3.4(4.0)	85.0%	6.4(8.0)	80.0%
$VMo_{0.3}Co_{0.7}COx$@NF	1.0/0.3/0.7	3.2(4.0)	80.0%	6.1(8.0)	76.3%

Table S2. Levels of independent variables for the response surface design for VMoCoCOx@NF with different mole ratios of Mo and Co

Independent variable	Factor level			
χ_1 (Mo, mol)	0.3	0.4	0.5	0.6
χ_2 (Co, mol)	0.4	0.5	0.6	0.7

The optimization equations of O_2 and H_2 prediction are as follows.

Or: Final equation in terms of coded factors:

(1) O_2: O_2 (%) = $+ 88.96 + 2.47\chi_1 - 2.20\chi_2 - 0.11\chi_1\chi_2 - 1.90\chi_1^2 - 2.95\chi_2^2$ (S1)

(2) H_2: H_2 (%) = $+ 91.54 + 6.37\chi_1 - 2.73\chi_2 - 1.55\chi_1\chi_2 - 6.47\chi_1^2 - 3.33\chi_2^2$ (S2)

Where, χ_1 and χ_2 are the coded forms of Mo mole amount and Co mole amount, respectively.

Table S3. Response surface optimal (custom) design and the experimental and predicted production of O_2 and H_2 by VMoCoCOx@NF with different ratios of Mo and Co.

Run	Factor		O_2 (%)		H_2 (%)	
	χ_1 (Mo, mol)	χ_2 (Co, mol)	experimental	predicted	experimental	predicted
1	0.6	0.5	89.80	89.96	93.90	92.51
2	0.4	0.4	86.50	87.13	87.60	87.59
3	0.3	0.7	80.00	79.55	76.30	74.20
4	0.6	0.7	84.30	84.26	83.40	83.84
5	0.5	0.6	88.90	88.49	93.10	91.50
6	0.4	0.6	85.00	86.87	80.00	87.59
7	0.6	0.7	84.30	84.26	83.40	83.84
8	0.6	0.4	87.50	88.88	85.50	92.41

9	0.4	0.6	85.00	86.87	80.00	87.59
10	0.3	0.7	80.00	79.55	76.30	74.20
11	0.3	0.5	85.50	84.96	78.70	78.73
12	0.3	0.5	85.50	84.96	78.70	78.73
13	0.5	0.5	92.50	89.98	96.30	93.66
14	0.3	0.4	83.20	83.72	76.80	76.56
15	0.5	0.6	88.90	88.49	93.10	91.50
16	0.5	0.4	90.40	88.85	94.20	92.87

Table S4. ANOVA for response surface quadratic model for O_2 production of VMoCoCOx@NF with different ratios of Mo and Co.

Source	Sum of squares	Degree of freedom	Mean square	F-value	P-value
Mode	159.78	5	31.96	18.34	< 0.0001
χ_1	57.08	1	57.08	32.76	0.0002
χ_2	41.37	1	41.37	23.74	0.0006
$\chi_1\chi_2$	0.079	1	0.079	0.045	0.8360
χ_1^2	9.08	1	9.08	5.21	0.0455
χ_2^2	23.20	1	23.20	13.31	0.0045
Residual	17.42	10	1.74		
Lack of fit	17.42	5	3.48		
Pure error	0.000	5	0.000		
Total	177.20	15			

Table S5. ANOVA for response surface quadratic model for H_2 production of VMoCoCOx@NF with different ratios of Mo and Co.

Source	Sum of squares	Degree of freedom	Mean square	F-value	P-value
Mode	735.42	5	147.08	27.78	< 0.0001
χ_1	381.28	1	381.28	72.01	< 0.0001
χ_2	63.93	1	63.93	12.07	0.0060
$\chi_1\chi_2$	15.61	1	15.61	2.95	0.1167
χ_1^2	105.21	1	105.21	19.87	0.0012
χ_2^2	29.41	1	29.41	5.55	0.0402
Residual	52.94	10	5.29		
Lack of fit	52.94	5	10.59		
Pure error	0.000	5	0.000		
Total	788.36	15			

Table S6. Optimum values for the ratio of Mo and Co for VMoCoCOx@NF and the production of O_2 and H_2.

Variable	Optimum value (χ_i)
V, mol	1

Mo, mol	0.538
Co, mol	0.483
O_2, %	90.170 (predicted)<br>91.621 ± 0.526[a] (experimental)
H_2, %	94.018 (predicted)<br>95.967 ± 0.395[a] (experimental)

[a] Mean ± standard deviation (*n* = 3).

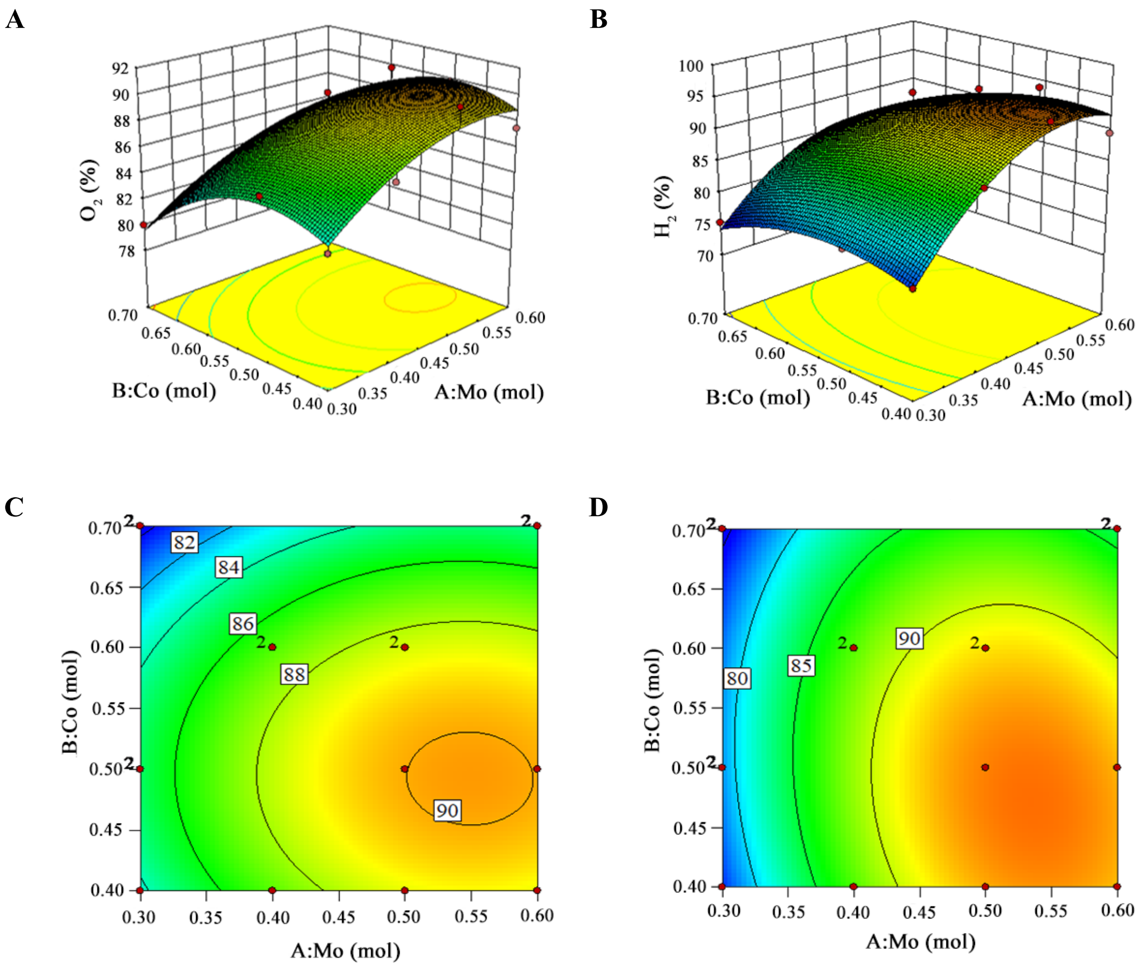

Figure S1. 3-D response surface plots and contour plots for the as-prepared catalysts with different mole ratios of Mo and Co. 3-D response surface plots for production of O_2 (A) and H_2 (B), contour plots for production of O_2 (C) and H_2 (D).

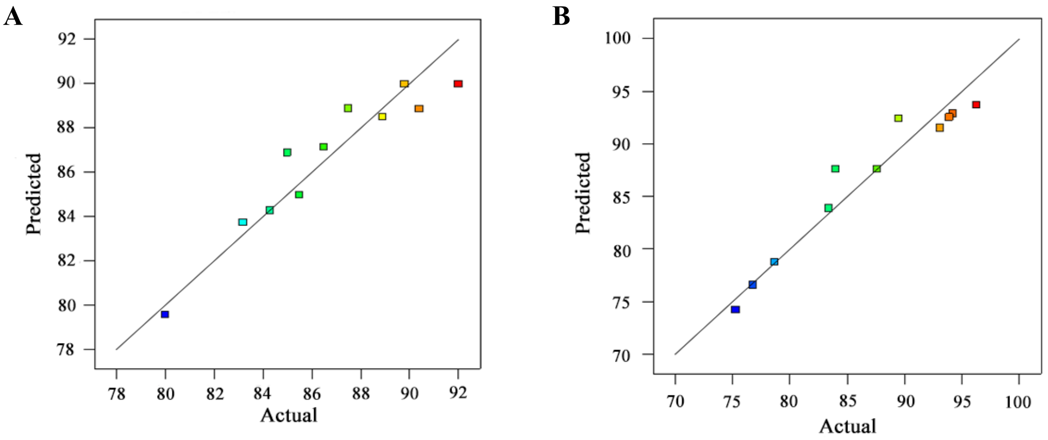

Figure S2. Fit plots for predicted and actual values. (A) O_2 production of VMoCoCOx@NF with different mole ratios of Mo and Co, R^2 = 0.9017, R^2 (adj) = 0.8525, R^2 (pred) = 0.7633, the "Pred R-Squared" of 0.7633 was in reasonable agreement with the "Adj R-Squared" of 0.8525 because the difference was less than 0.2; (B) H_2 production of VMoCoCOx@NF with different mole ratios of Mo and Co, R^2 = 0.9328, R^2 (adj) = 0.8993, R^2 (pred) = 0.8258, the "Pred R-Squared" of 0.8258 was in reasonable agreement with the "Adj R-Squared" of 0.8993 because the difference was less than 0.2.

By comparing the model prediction and the actual data, the adequacy of RSM model could be comprehend, as shown in Fig. S2. The acceptable agreement between them indicated that the obtained model could perform well in predicting and describing the optimized mole ratio of designed catalyst. It was now possible to predict optimal formulation parameters based on the RSM model, which the obtained results were given in Table S6.

2. Additional Experimental Details

2.1 Chemicals and Materials

All the chemicals were of analytical grade or better, and used as received without further purification. Ammonium metavanadate (NH_4VO_3), cobaltous chloride hexahydrate ($CoCl_2 \cdot 6H_2O$), ammonium molybdate(VI)[$(NH_4)_2MoO_4$], potassium hydroxide (KOH), sodium hydroxide (NaOH), hydrochloric acid (HCl), ammonium persulfate [$(NH_4)_2S_2O_8$], and urea (CH_4N_2O) were purchased from Sigma Aldrich, Shanghai, China. Nickel foam supplied by MTI Corporation, with a thickness of 1.5

mm and over 99% purity, was used as substrate. Deionized (DI) water (18 MΩ cm^{-1}) was used in electrochemical measures and hydrothermal deposition preparation.

2.2 Density Functional Theory (DFT)

DFT calculation was performed using Vienna ab initio Simulation Package (VASP) with the generalized gradient approximation (GGA) parameterized by Perdew, Burke and Ernzerhof (PBE) for the exchange correlation function. Energy cut off of 500 eV and applicable k-points of 2 × 1 × 1 were used. Besides, systemic energy tolerance of 1×10^{-5} eV and remaining total force of 10^{-2} eV $Å^{-1}$were also employed.

2.2.1 Alkaline Electrolyte (KOH 1.0 mol L^{-1})

Based on the standard hydrogen electrode (SHE) model, in alkaline media, the HER process can be divided into three steps (HER half-reaction: $2H_2O + 2e^- \rightarrow H_2\uparrow + 2OH^-$) [1]:

2.2.1.1 HER on the cathode in alkaline electrolyte (KOH 1.0 mol L^{-1})

$* + H_2O + e^- \rightarrow H^* + OH^-$ (Volmer Reaction) (S3)

$H^* + H_2O + e^- \rightarrow H_2\uparrow + OH^-$ (Heyrovsky Reaction) (S4)

or $H^* + H^* \rightarrow H_2\uparrow$ (Tafel Reaction) (S5)

where the star * represents the adsorption site, and the term of H* is the adsorbed intermediate on the active site of a catalyst. Both pathways involve the adsorption of H_2O, electrochemical reduction into adsorbed H atom and OH^-, and desorption of H_2. The formula for H* adsorption on the surface of catalysts and the calculation of Gibbs free energy (ΔG_{H^*}) for HER are listed follows.

$$\Delta E_{H^*} = E(_{H^*Slab}) - E(S_{lab}) - 0.5E_{(H2)} \quad (S6)$$

$$\Delta G_{H^*} = \Delta E_{H^*} + \Delta E_{ZPE} - T\Delta S \quad (S7)$$

where $E_{(H^*Slab)}$ is the energy of the total system with absorbing individual hydrogen atoms, $E_{(Slab)}$ is pure surface energy, $E_{(H2)}$ is hydrogen molecule energy, and ΔE_{ZPE} is the zero-point energy and ΔS is the change of entropy, T = 298.15 K.

2.2.1.2 OER on the cathode in alkaline electrolyte (KOH 1.0 mol L^{-1})

The OER in alkaline media is the four-elctron-transfer process with an overall reaction (OER half-reaction: $4OH^{-} \rightarrow O_2\uparrow + 2H_2O + 4e^{-}$).

$* + OH^{-} \rightarrow OH^* + e^{-}$ (S8)

$OH^* + OH^{-} \rightarrow O^* + H_2O + e^{-}$ (S9)

$O^* + OH^{-} \rightarrow OOH^* + e^{-}$ (S10)

$OOH^* + OH^{-} \rightarrow {}^*O_2 + H_2O + e^{-}$ (S11)

${}^*O_2 \rightarrow * + O_2\uparrow$ (S12)

where the star * represents the adsorption site. The Gibbs free energy (ΔG) of these adsorption oxygen-containing intermediates (OH*, O*, OOH*) is calculated by the formula of

$\Delta G = \Delta E + \Delta E_{ZPE} - T\Delta S$ (S13)

Herein, ΔE, ΔE_{ZPE}, and TΔS are the DFT calculated total energy differences, the difference between zero-point energy change and change of entropy, respectively.

For above each reaction step, the Gibbs free energy can obtained using the above-mentioned ΔG equation at T = 298.15 K. In the case of OER, when electrode potential U = 0 V, the Gibbs free energy change for each elementary step (ΔGi, i = 1, 2, 3, 4) is calculated as following equations.

$\Delta G_1 = \Delta G_{OH^*}$ (S14)

$\Delta G_2 = \Delta G_{O^*} - \Delta G_{OH^*}$ (S15)

$\Delta G_3 = \Delta G_{OOH^*} - \Delta G_{O^*}$ (S16)

$\Delta G_4 = 4.92\ eV - \Delta G_{OOH^*}$ (S17)

The theoretical overpotentials (η_{HER} and η_{OER}), which are the measure of catalytic performance, can be estimated for HER and OER as follows:

$\eta_{HER} = |\Delta G_{H^*}|/e$ (S18)

$\eta_{OER} = |\max(\Delta G_1, \Delta G_2, \Delta G_3, \Delta G_4)/e| - 1.23\ V$ (S19)

2.2.2 Acidic Electrolyte (H_2SO_4 0.5 mol L^{-1})

The specific reaction pathways in acidic medium are shown as follows [2].

2.2.2.1 HER on the cathode in acidic electrolyte (H_2SO_4 0.5 mol L^{-1}) (HER

half-reaction: $2H^+ + 2e^- \rightarrow H_2\uparrow$)

$H^+ + e^- + * \rightarrow H^*$ (Volmer Reaction) (S20)

$H^* + H^+ + e^- \rightarrow H_2\uparrow$ (Heyrovsky Reaction) (S21)

or $H^* + H^* \rightarrow H_2\uparrow$ (Tafel Reaction) (S22)

$\Delta E_{H^*} = E_{(H^*Slab)} - E(S_{lab}) - 0.5E_{(H2)}$ (S23)

$\Delta G_{H^*} = \Delta E_{H^*} + \Delta E_{ZPE} - T\Delta S$ (S24)

When HER occurs in the acidic electrolyte, Volmer reaction occurs first. At the adsorption site on the electrode surface, a proton H^+ from the electrolyte will combine with an electron e^- from the electrode surface, thus forming an adsorbed hydrogen H* on the electrode surface.

2.2.2.2 OER on the anode in acidic electrolyte (H_2SO_4 0.5 mol L^{-1}) (OER half-reaction: $2H_2O \rightarrow O_2\uparrow + 4H^+ + 4e^-$)

$H_2O + * \rightarrow {}^*OH + H^+ + e^-$ (S25)

$^*OH \rightarrow {}^*O + H^+ + e^-$ (S26)

$^*O + H_2O \rightarrow {}^*OOH + H^+ + e^-$ (S27)

$^*OOH \rightarrow {}^*O_2 + H^+ + e^-$ (S28)

$^*O_2 \rightarrow * + O_2\uparrow$ (S29)

$\Delta G_1 = \Delta G_{OH^*}$ (S30)

$\Delta G_2 = \Delta G_{O^*} - \Delta G_{OH^*}$ (S31)

$\Delta G_3 = \Delta G_{OOH^*} - \Delta G_{O^*}$ (S32)

$\Delta G_4 = 4.92\ eV - \Delta G_{OOH^*}$ (S33)

To summarize, ΔE_{H^*}, ΔG_{H^*} and ΔG1–ΔG4 are independent of pH, and therefore they are applicable to water-acidic conditions.[4] The intermediates for OER in acidic or alkaline electrolytes are the same, forming *OH, *O, *OOH and $^*O_2$. The Gibbs free energy changes for each elementary step (ΔGi, i = 1, 2, 3, 4) are usually used to represent the four intermediates, among which the η_{OER} is also commonly used to evaluate the reaction process of OER. The maximum step in the ΔG value is usually the decisive step in the OER process, often occurring in the step from *OH to *O, or

from *O to *OOH. The theoretical onset overpotentials of HER and OER on the catalyst are determined by the equations (S18) and (S19).

3. Experimental Figures and Tables

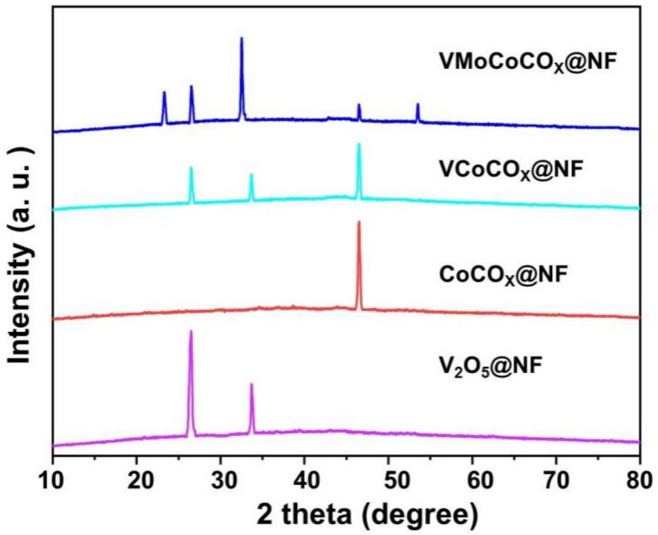

Figure S3. XRD patterns of VMoCoCOx@NF, VCoCOx@NF, CoCOx@NF and V_2O_5@NF on the same coordinates. Note: the diffraction lines of Ni were removed during XRD operation.

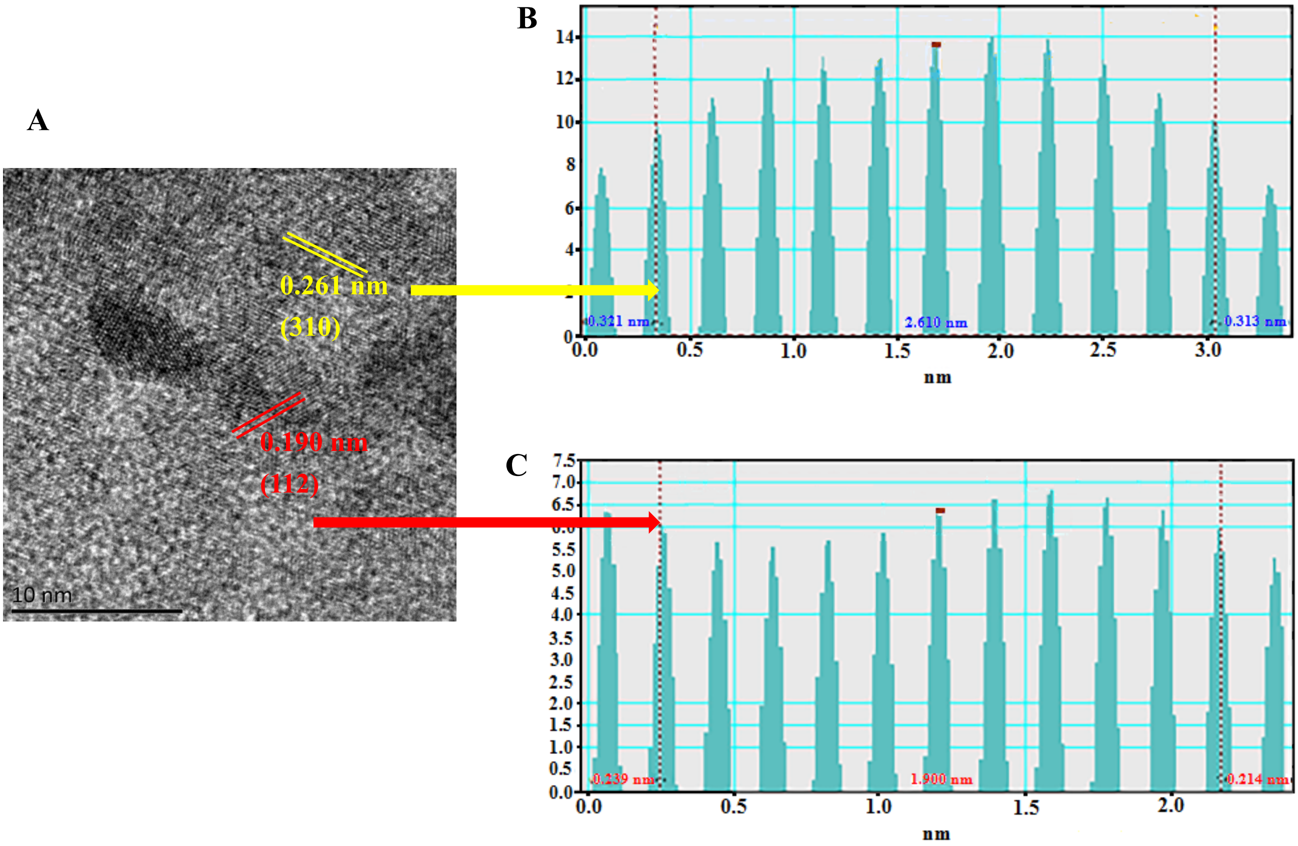

Figure S4. (A) HRTEM image of VMoCoCOx@NF. (B) The lattice spacing

distributions corresponding to 0.261 nm for the (310) plane and (C) 0.190 nm for the (112) plane.

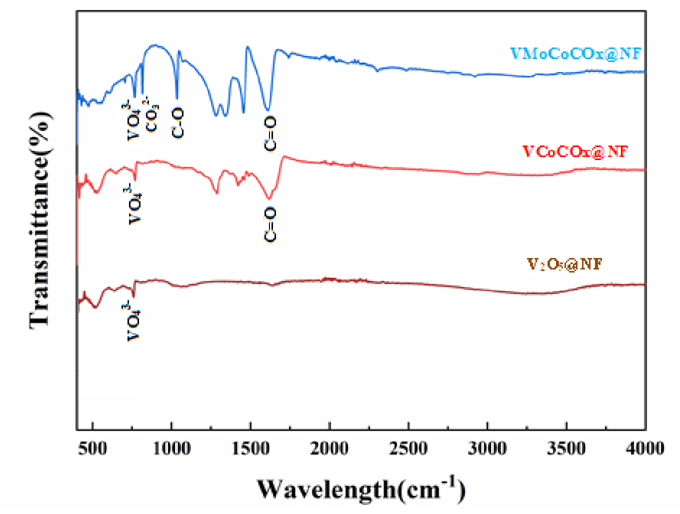

Figure S5. The FTIR spectra of VMoCoCOx@NF, VCoCOx@NF and V_2O_5@NF.

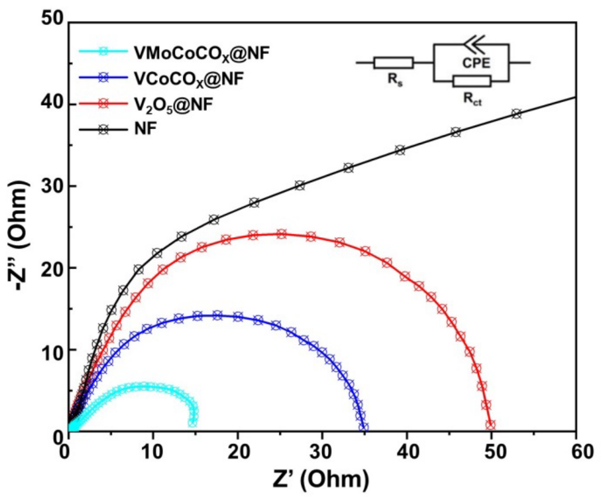

Figure S6. Nyquist plots of HER for the four catalytic electrodes, and the inset is the equivalent circuit model.

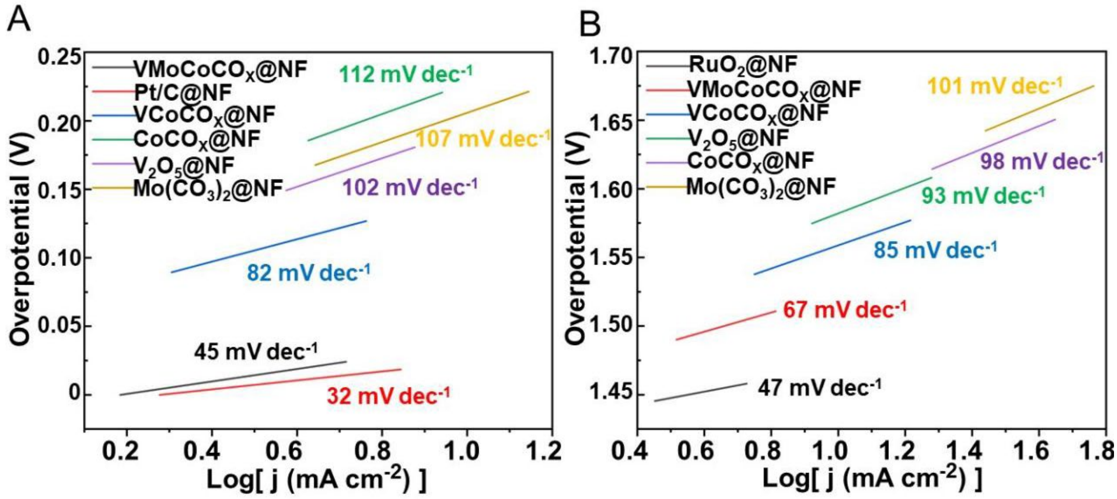

Figure S7. Comparison of Tafel slopes of the catalysts selected for HER (A) at a constant $j = -10$ mA cm^{-2} and OER (B) at a constant $j = 10$ mA cm^{-2}.

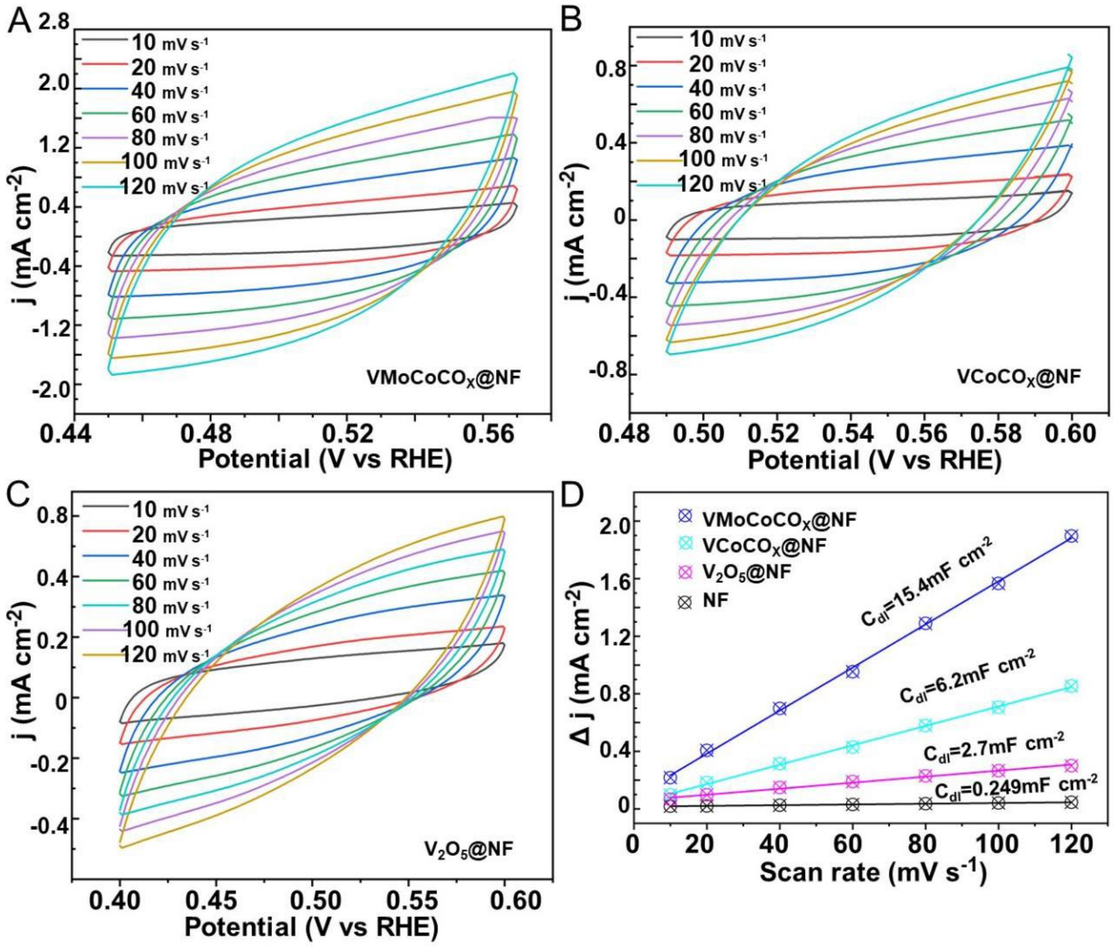

Figure S8. The original CV curves of VMoCoCOx@NF (A), VCoCOx@NF (B) and V_2O_5@NF (C) recorded at various scan rates (10, 20, 40, 60, 80, 100, and 120 mV s^{-1}) in the non-faradaic region for the estimation of C_{dl}. (D) Estimation of C_{dl} values of VMoCoCOx@NF, VCoCOx@NF, CoCOx@NF and V_2O_5@NF by plotting the ratios of catalyst ΔJ to scan rate.

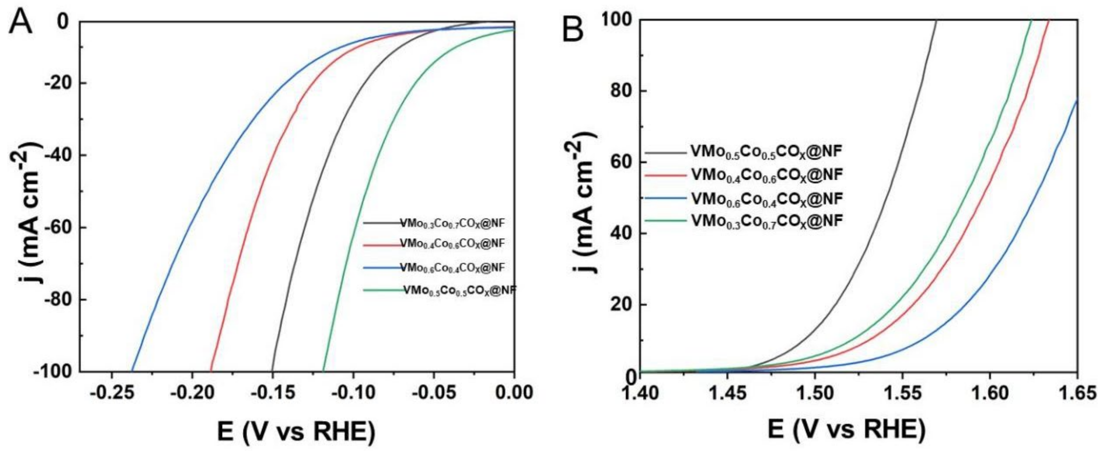

Figure S9. The polarization curves of $VMo_{0.5}Co_{0.5}COx$@NF, $VMo_{0.6}Co_{0.4}COx$@NF, $VMo_{0.4}Co_{0.6}COx$@NF, and $VMo_{0.3}Co_{0.7}COx$@NF for HER (A) and OER (B).

A: XRD

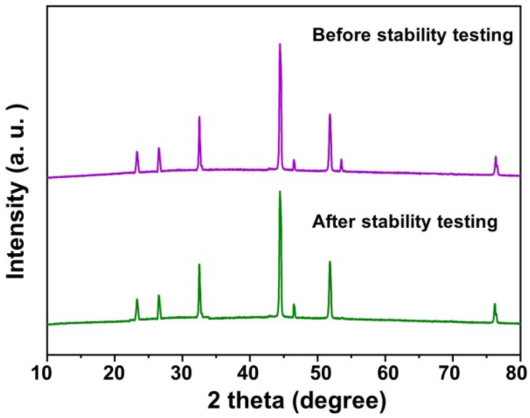

B: SEM

Before stability testing

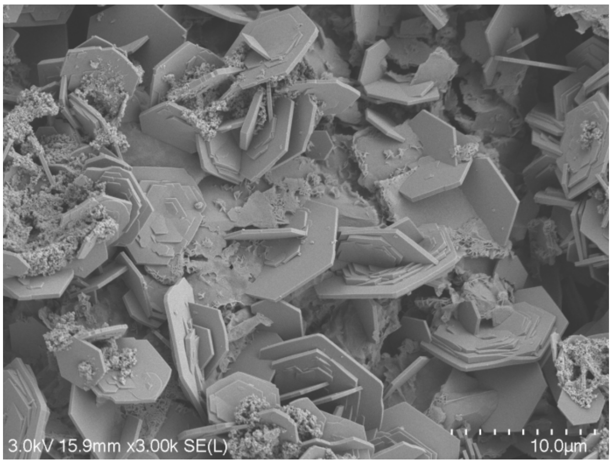

After stability testing

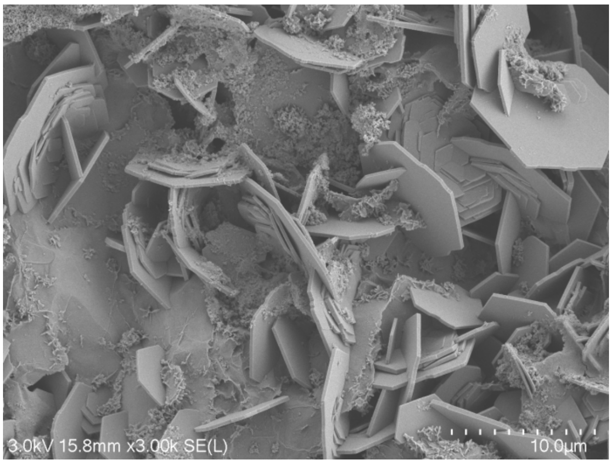

C: XPS: (A) before stability testing; (B)after stability testing.

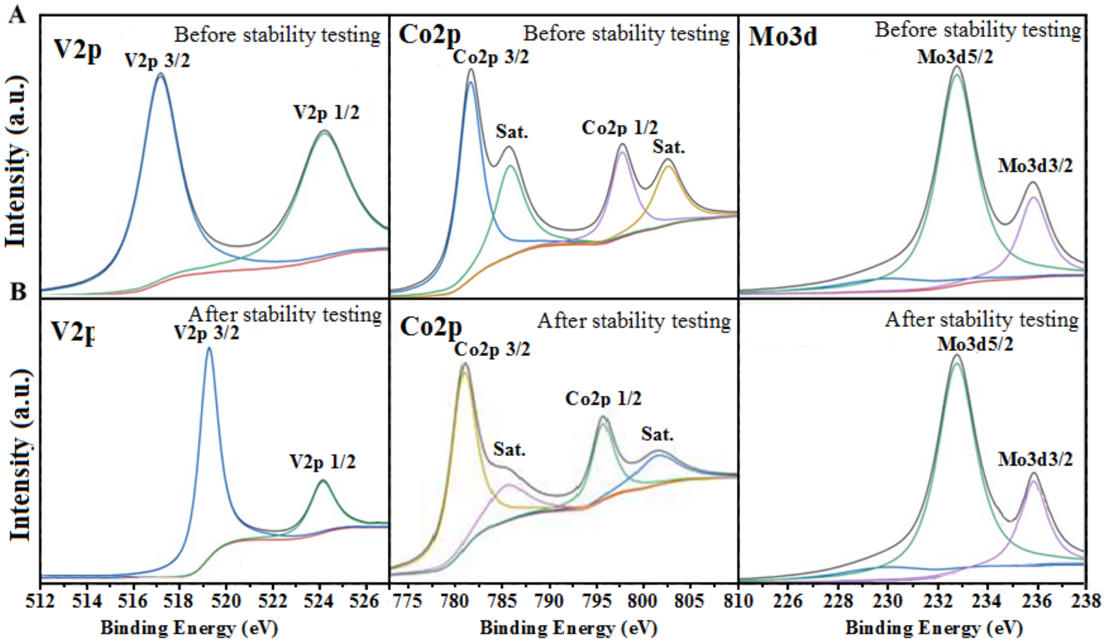

Figure S10. The structure (XRD)(A), morphology (SEM)(B) and chemical state (XPS) (C) of the sample before and after stability testing.

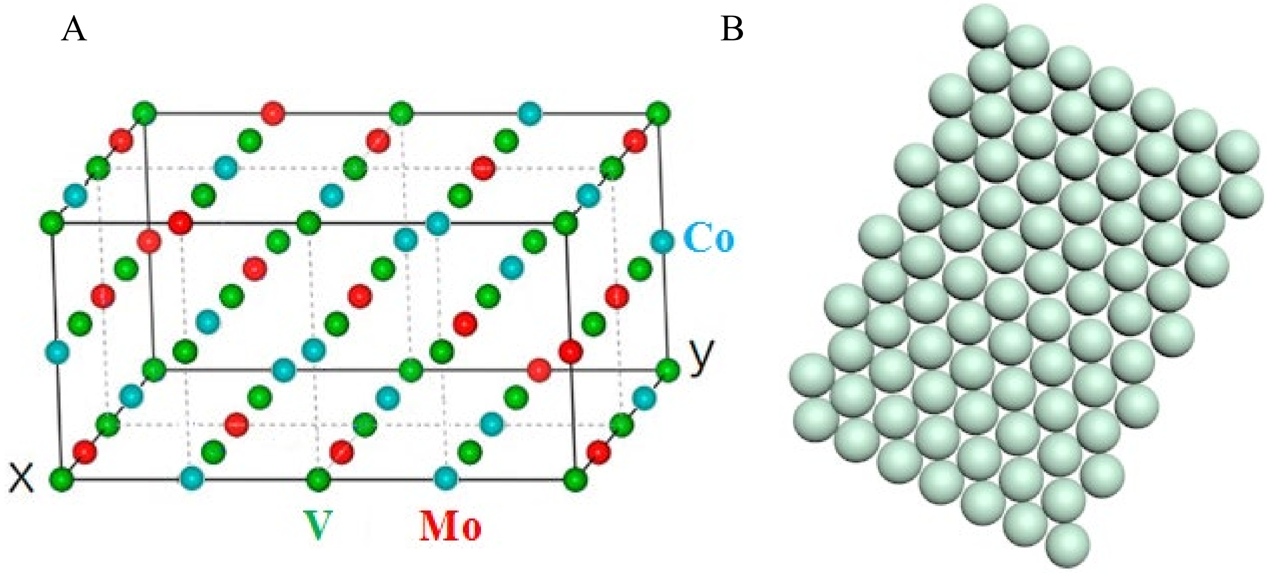

Figure S11. (A) The crystal plane of VMoCoCOx (112); The Mo (Red ball) and V sites (green ball) share the same position in the crystal structure and are bridged by Co (blue ball). (B) Crystal structure of NF (111) in top view.

Table S7. XRD data of the target materials.

Data	Target	2θ (°)
PDF# database	MoO_3 (PDF#05-0508)	23.3°, 25.7°, 27.3°
	$CoCO_3$ (PDF#11-0692)	42.9º, 46.5º, 51.8º, 53.5º.
	V_2O_5 (PDF#00-041-1426)	19.88º, 26.5º(110), 30.8º, 33.7º, 47.1º, 50.8º.
	Ni (PDF#04-0850)	44.2°, 51.8°, 76.3°.
Experimental data	VMoCoCOx@NF	23.3°(MoO_3), 26.5º(V_2O_5), 32.5°(New phase), 44.2º, 46.5º($CoCO_3$), 51.8º, 53.5º, 76.3º
	VCoCOx@NF	26.5º(V_2O_5), 33.7º(V_2O_5), 44.2º, 46.5º($CoCO_3$), 51.8º, 76.3º.
	CoCOx@NF	44.2°, 46.5º($CoCO_3$), 51.8°, 76.3°
	V_2O_5@NF	26.5º(V_2O_5), 33.7º, 44.2°, 51.8°, 76.3°

Table S8. Comparison of the recently reported NF-supported transition metallic electrocatalysts for the electrocatalytic activities (HER, OER, and overall water splitting) in 0.1M KOH electrolyte.

**Electrocatalyst**	**HER (η_{10})**		**OER (η_{10})**		**Over water splitting**		**Reference**
	**Overpotential (mV)**	**Tafel slope (mV dec^{-1})**	**Overpotential (mV)**	**Tafel slope (mV dec^{-1})**	**Voltage (η_{10}) (V vs. RHE)**	**Voltage (η_{100}) (V vs. RHE)**	
$V_2O_5/Ni(OH)_2/NF$	39	44	360	52	1.59	−	[3]
$CoP_3/NiMoO_4/NF$	92	56	347	69.7	1.57	−	[4]
$NiO–CoFe_2O_4/NF$	97	89	157	82	1.54	−	[5]
$Mo-Co_3O_4$@NF	79	98	315	83.7	1.64	−	[6]
$\beta-Ni(OH)_2/NF$	170	51	329	40	1.73	−	[7]
VCoCOx@NF	63	93	240	65	1.54	1.74	[8]
$PA@CuCo_2S_4/NF$	107	85.9	110	142.7	1.55	−	[9]
$Mo-Co(OH)_2/Co_3O_4/NF$	116	111	234	128.6	1.62	−	[10]
$NiMn_2O_4/NF$	248	198	250	218	1.69	-	[11]
VMoCoCOx@NF	37	45	264	67	1.54	1.61	This work

η_{10}: 10 mA cm^{-2} current density; η_{100}: 100 mA cm^{-2} current density.

−: Not tested.

References

1. Zou, W.J.; Xiang, J.D.; Tang, H. Niobium-doped cobalt phosphide nanowires realizing enhanced electrocatalytic activity for overall water splitting. *Int. J. Hydrogen Energ.* **2022**, 47, 13251−13260. [CrossRef]
2. Li, S.B.; B. Fei, Two-dimensional transition metal-based electrocatalyst and their application in water splitting. *Mater. Sci. Tech-Lond.* **2022**, 38, 535–555. [CrossRef]
3. Meena, A.; Ha, M.; Chandrasekaran, S.S.; Sultan, S.; Thangavel, P.; Harzandi, A.M.; Singh, B.; Tiwari, J.N.; Kim, K.S. Pt-like hydrogen evolution on a $V_2O_5/Ni(OH)_2$ electrocatalyst. *J. Mater. Chem. A* **2019**, 7, 15794. [CrossRef]
4. Wang, Y.Q.; Zhao, L.; Sui, X.L.; Gu, D.M.; Wang, Z.B. Hierarchical $CoP_3/NiMoO_4$ heterostructures on Ni foam as an efficient bifunctional electrocatalyst for overall water splitting. *Ceram. Int.* **2019**, 45, 17128–17136. [CrossRef]
5. Wang, X.F.; Liu, Z.H.; Guo, Z.G.; Ge, L.; Liu, Z.F. NiO-$CoFe_2O_4$ electrocatalyst prepared on Ni foam by one-step hydrothermal method for efficient overall water splitting. *J. Mater. Sci.* **2021**, 56, 8575–8587. [CrossRef]
6. Xiong, S.S.; Weng, S.T.; Tang, Y.; Qian, L.; Xu, Y.Q.; Li, X.F.; Lin, H.J.; Xu, Y.C.; Jiao, Y.; Chen, J.R. Mo-doped Co_3O_4 ultrathin nanosheet arrays anchored on nickel foam as a bi-functional electrode for supercapacitor and overall water splitting. *J. Colloid Interface Sci.* **2021**, 602, 355–366. [CrossRef]
7. Yang, J.H.; Xu, X.H.; Chen, M.M.; Yang, D.; Lu, H.; Sun, Y.Z.; Shao, C.; Song, Q.Q.; Zhang, J.; Gao, L.; Zhang, Y.T. Morphology-controllable nanocrystal β-$Ni(OH)_2$/NF designed by hydrothermal etching method as high-efficiency electrocatalyst for overall water splitting. *J. Electroanalytical Chem.* **2021**, 882, 115035. [CrossRef]
8. Meena, A.; Thangavel, P.; Nissimagoudar, A.S.; Singh, A.N.; Jana, A.; Jeong, D.S.; Im, H.; Kim, K.S. Bifunctional oxovanadate doped cobalt carbonate for high-efficient overall water splitting in alkaline-anion-exchange-membrane water-electrolyzer. *Chem. Eng. J.* **2022**, 430, 132623. [CrossRef]
9. Shen, Y.J.; Chen, Y.; Fang, S.K.; Park, J.K.; Feng, K. Plasma-modified Ni foam-supported $CuCo_2S_4$ nanowires as bifunctional electrocatalysts for high-performance overall water splitting. *Arabian J. Chem.* **2023**, 16, 104989. [CrossRef]
10. Yang, H.; Hu, T.P.; Meng, R.Q.; Guo, L.J. Efficient Mo-$Co(OH)_2/Co_3O_4$/Ni foam electrocatalyst for overall water splitting. *J. Solid State Chem.* **2023**, 320, 123837. [CrossRef]
11. Nagajyothi, P.C. ; Pavani, K. ; Ramaraghavulu, R. ; Shim, J. Microwave synthesis of $NiMn_2O_4$/Ni-foam: Efficient bifunctional electrocatalysts for overall water splitting. *Int. J. Hydrogen Energ.* **2023**, 54, 691−699. [CrossRef]
